# Nanoparticles modulate autophagic effect in a dispersity-dependent manner

**DOI:** 10.1038/srep14361

**Published:** 2015-09-23

**Authors:** Dengtong Huang, Hualu Zhou, Jinhao Gao

**Affiliations:** 1 The Key Laboratory for Chemical Biology of Fujian Province, State Key Laboratory of Physical Chemistry of Solid Surfaces, and Department of Chemical Biology, College of Chemistry and Chemical Engineering, Xiamen University, Xiamen 361005, China

## Abstract

Autophagy plays a key role in human health and disease, especially in cancer and neurodegeneration. Many autophagy regulators are developed for therapy. Diverse nanomaterials have been reported to induce autophagy. However, the underlying mechanisms and universal rules remain unclear. Here, for the first time, we show a reliable and general mechanism by which nanoparticles induce autophagy and then successfully modulate autophagy *via* tuning their dispersity. Various well-designed univariate experiments demonstrate that nanomaterials induce autophagy in a dispersity-dependent manner. Aggregated nanoparticles induce significant autophagic effect in comparison with well-dispersed nanoparticles. As the highly stable nanoparticles may block autophagic degradation in autolysosomes, endocytosis and intracellular accumulation of nanoparticles can be responsible for this interesting phenomenon. Our results suggest dispersity-dependent autophagic effect as a common cellular response to nanoparticles, reveal the relationship between properties of nanoparticles and autophagy, and offer a new alternative way to modulate autophagy.

Autophagy is an evolutionarily conserved and lysosome dependent protein degradation pathway^1^. During autophagy, a double-membrane structure engulfs protein aggregates, damaged organelles and other cellular components to create autophagosome. Then the autophagosome fuses with lysosome and forms an autolysosome. Subsequently, the inner membrane and inside components are degraded by lysosomal hydrolases and released to cytosolic space^2^. Autophagy runs in a basal level to maintain cellular homeostasis while up-regulates under stress conditions (*e.g.*, nutrient deprivation, oxidative stress and hypoxia) to help cells survive^3^,^4^. It is associated with many human diseases^5^,^6^ so that autophagy regulation has become a new therapeutic strategy^7^,^8^. Many compounds such as rapamycin, resveratrol and torin1 have been developed for autophagy regulation^9^,^10^.

Recently, a great diversity of nanoparticles (*e.g.*, rare earth oxide, quantum dots, iron oxide, gold, silica, titanium dioxide and carbon) have been reported to induce autophagy^11^,^12^. Nanoparticles are considered to be a new class of autophagy activators^13^,^14^ and combined with chemical drugs in cancer therapy^15^,^16^. However, the underlying mechanism remains unclear. We attempt to reveal the answer. In this study, we used iron oxide (IO) nanoparticles as a major subject because of their widespread use and excellent biocompatibility^17^,^18^. Our results showed that IO nanoparticles induced significant autophagic effect when they were aggregated. However, if we improved the dispersity of the same materials by surface modification or protein adsorption, they showed little autophagic effect. Moreover, other promising nanoparticles, such as gold and silica nanoparticles, likewise, exhibited dispersity-dependent autophagic effect. These results demonstrated that we could modulate autophagy by nanoparticles through tuning their dispersity.

## Results

**Figure 1:**
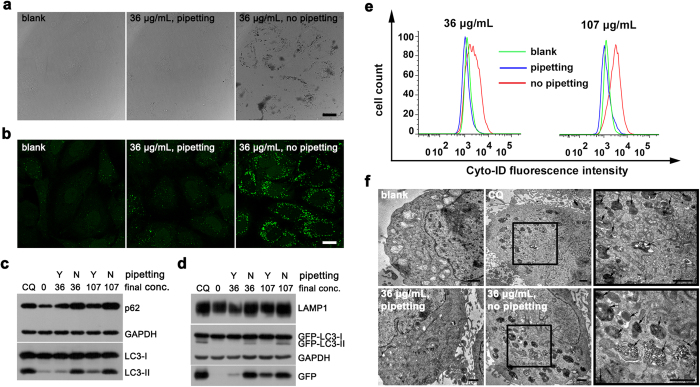
IO@citrate nanoparticles aggregations induce accumulation of autophagic vacuoles. (**a**) Confocal bright field images and (**b**) Fluorescence images of HeLa-GFP-LC3 cells after treatment for 24 h. (**c**,**d**) Western blot analysis of autophagy-related proteins after treatment. Cells treated with 10 μM chloroquine (CQ) as a positive control, and cells without any treatment as a negative control. (**e**) Flow cytometry quantification of autophagic vacuoles. (**f**) TEM images of HeLa-GFP-LC3 cells before and after treatments. The right views were the magnification of the region with a black box in the middle views. The black arrows in the right views pointed to autophagic vacuoles. Scale bars were 20 μm (**a**,**b**) and 1 μm (**f**) respectively.

We incubated the same dose of IO nanoparticles (coated with sodium citrate, indicated as IO@citrate) with HeLa cells that stably express GFP-LC3 (green fluorescent protein fused with microtubule-associated protein 1 light chain 3) in two groups. However, one group showed significant autophagic effect while the other was not (Fig. 1). The only difference was whether we pre-mixed nanoparticles with complete medium by pipetting (pipetting group) or not (no-pipetting group). In former case, there was little aggregation of nanoparticles. In latter case, plenty of nanoparticles aggregated and precipitated on the surface of cells, which could be visualized by scanning electron microscopy (SEM) (Fig. 1a and Supplementary Figs S1, S2). Aggregated nanoparticles elicited plenty of GFP-LC3 puncta compared to blank and pipetting groups (Fig. 1b). Chloroquine (CQ), which can raise the pH of lysosome and cause blocked autophagy^19^, showed similar results. Immunofluorescence revealed that most GFP-LC3 puncta were collocated with p62 puncta (Supplementary Fig. S3), which is a characteristic of autophagic vacuoles^20^. Western blot confirmed the conversion of endogenous LC3-I to LC3-II (Fig. 1c), a marker of autophagosome^21^, and the conversion of GFP-LC3-I to GFP-LC3-II (Fig. 1d), which has the similar behaviors to LC3-II^22^. p62, a special autophagic substrate protein^23^, and GFP fragment, a partial degradation product of GFP-LC3 in autolysosome^24^, were up-regulated. Moreover, lysosomal-associated membrane protein 1 (LAMP1), a marker of lysosome^25^, was also up-regulated, indicating an increase of lysosome or autolysosome. Up-regulation of these autophagy-related proteins revealed that aggregated nanoparticles caused accumulation of autophagic vacuoles. Of course, this phenomenon could be diminished by 3-methyladenine (3-MA, Supplementary Fig. S4), an autophagy inhibitor^19^.

We engaged Cyto-ID^TM^ Autophagy Detection Kit to quantify the autophagic vacuoles. This commercial kit is special for detection of autophagic vacuoles while with negligible staining of lysosomes^26^. The fluorescence intensity of Cyto-ID in no-pipetting groups (aggregated) was much higher than that in pipetting (well-dispersed) and blank groups (Fig. 1e). This result further revealed that aggregated nanoparticles elicited much more autophagic vacuoles than well-dispersed nanoparticles.

Transmission electron microscopy (TEM) is the gold standard for autophagy detection^19^. We could observe massive autophagic vacuoles in CQ-treated and no-pipetting groups, while little in pipetting and blank groups (Fig. 1f and Supplementary Fig. S5), demonstrating that aggregated nanoparticles elicited much autophagic vacuoles. Moreover, there were much more nanoparticles inside cells in no-pipetting group than in pipetting group, suggesting that endocytosis amount of nanoparticles may be a key factor in autophagic effect. Investigation by western blot and immunofluorescence revealed that aggregation of nanoparticles induced accumulation of autophagic vacuoles by time and dose dependence (Supplementary Figs S6, S7). Additionally, we observed similar autophagic effect mediated by nanoparticles in SKOV3 cells stably expressed GFP-LC3 (SKOV3-GFP-LC3) (Supplementary Figs S8, S9). Consequently, aggregated nanoparticles induced accumulation of autophagic vacuoles while well-dispersed nanoparticles did not.

**Figure 2:**
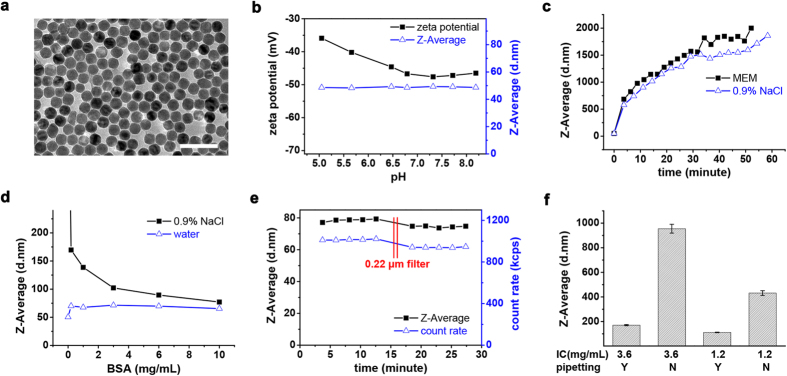
Protein adsorption reduces aggregation of nanoparticles in saline. (**a**) TEM image of IO@citrate nanoparticles dispersed in water. Scale bar was 100 nm. (**b**) Hydrodynamic diameter and zeta potential of IO nanoparticles over pH range. (**c**–**f**) Hydrodynamic diameter of IO nanoparticles dispersed in various media. (**c**) MEM or 0.9% NaCl. (**d**) BSA aqueous or saline solution. (**e**) 10 mg/mL BSA saline solution. Solution was filtered by 0.22 μm filter at 16 minutes time point. (**f**) Complete medium. IO nanoparticles with various initial concentrations (IC) were mixed with complete medium by pipetting or not. The final concentration of Fe was 36 μg/mL (*n* = 4. Data represent mean ± s.d.).

We next investigate why nanoparticles aggregated in no-pipetting group. The 27 nm IO nanoparticles were synthesized from thermolysis of iron oleate and then transferred into water by sodium citrate coating (Fig. 2a). IO@citrate nanoparticles are quite stable in water. They kept their zeta potential below −30 mV and size (indicated as Z-Average) at about 48 nm in a range of pH 5 to 8 (Fig. 2b). Although IO@citrate nanoparticles dispersed well in water with long-term stability, they quickly aggregated and eventually formed micro-sized particles in physiological saline or minimum essential medium (MEM) without fetal bovine serum (FBS) (Fig. 2c). However, protein (*e.g.*, bovine serum albumin, BSA) adsorption on nanoparticles prevented their aggregation^27^,^28^. As the concentration of BSA increased to 10 mg/mL, the particle size of IO@citrate in saline dramatically decreased from >500 nm to 77 nm. This was closed to the size of 70 nm in water (Fig. 2d) and enabled the solution to pass 0.22 μm filter without notable decreasing in count rate of scattered photon (Fig. 2e), indicating that BSA almost abolished aggregation of IO@citrate nanoparticles in saline.

Generally, nanoparticles are dispersed in media containing both electrolytes and proteins for cell study. Electrolytes make nanoparticles aggregated whereas protein adsorption prevents it. Thus their relative rate eventually determines the disperse state of nanoparticles. Concentrated proteins accelerate protein adsorption. Similarly, mixing quickly by pipetting or diluting nanoparticles will accelerate diffusion and augment protein adsorption, resulting in diminished aggregation. Conversely, concentrated nanoparticles or mixing by spontaneous diffusion will slow down protein adsorption and enhance aggregation. For example, when dispersing IO@citrate nanoparticles in complete medium, we decreased the size of aggregation from micro-size to 170 nm by pipetting, and further reduced it to 110 nm by diluting nanoparticles (Fig. 2f). This study offered at least two methods to diminish aggregation of nanoparticles in complete medium: pipetting and dilution. Furthermore, other methods which enhance protein adsorption will certainly improve the dispersity of nanoparticles. These enable us to control the dispersity of nanoparticles (*e.g.*, aggregated or not, and aggregation size) as a single variable and eliminate other variables. We have adopted pipetting to tune the dispersity of nanoparticles and then to modulate autophagy above. Other methods will be examined for the ability to modulate autophagy.

**Figure 3:**
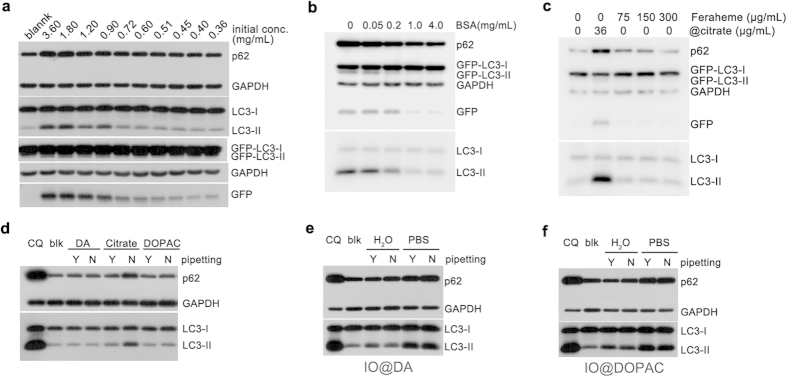
Modulating autophagy by IO nanoparticles with various surface chemistry. (**a**) IO@citrate nanoparticles were diluted before incubating with cells. Nanoparticles were added by no pipetting at different initial concentrations but the same final concentration. (**b**) IO@citrate nanoparticles pre-incubated with various concentrations of BSA aqueous solution and then added to medium without pipetting. (**c**) Feraheme did not elicited autophagic effect because of its excellent dispersity. (**d**) IO@DA and IO@DOPAC nanoparticles exhibited low autophagic effect compared to IO@citrate nanoparticles. (**e**) IO@DA nanoparticles (**f**) IO@DOPAC nanoparticles caused accumulation of autophagic vacuoles when they were dispersed in PBS. All cells were treated for 24 h. blk: blank, cells without any treatment.

Besides pipetting, dilution alters the dispersity of nanoparticles in complete medium as well. We diluted nanoparticles with water to augment protein adsorption. As shown in the bright field images, dilution dramatically diminished precipitation of nanoparticles and thus reduced GFP-LC3 puncta (Supplementary Fig. S10). Autophagy-related proteins, including LC3-II, GFP-LC3-II, GFP fragment and p62, were down-regulated as dilution times increased (Fig. 3a). Similarly, incubating nanoparticles with BSA would augment protein adsorption and improve dispersity, followed by alteration in autophagic effect. Actually, BSA with the concentration of >0.2 mg/mL could diminish the aggregation and precipitation of nanoparticles considerably (Supplementary Fig. S10) and result in notable reduction of autophagic effect (Fig. 3b). These results suggested that dilution and BSA incubation also improved the dispersity of nanoparticles and therefore diminished autophagic effect.

Surface chemistry of nanoparticles is important to autophagic effect^29^,^30^. We modified the surface of IO nanoparticles with various ligands (Supplementary Fig. S11), for example, dopamine (DA), 3,4-dihydroxyphenylacetic acid (DOPAC), and meso-2,3-dimercaptosuccinicacid (DMSA). Although these IO nanoparticles have high zeta potential and are stable in water (Supplementary Table S1), they show distinct stability in saline: IO@DMSA > IO@DA > IO@DOPAC > IO@citrate (Supplementary Fig. S12). The diverse stability in saline may affect autophagic response towards these materials.

Actually, whether mixed IO@DA or IO@DOPAC nanoparticles with complete medium by pipetting or not, little precipitation was observed on the surface of cells (Supplementary Fig. S13), and LC3-II level was closed to negative control accordingly (Fig. 3d). Similarly, IO@DMSA nanoparticles were dispersed well in complete medium and did not cause accumulation of autophagic vacuoles (Supplementary Fig. S14). Another type of IO nanoparticles, Feraheme was dispersed well in physiologic media and did not stimulate autophagy even with a concentration of 300 μg/mL (Fig. 3c and Supplementary Fig. S13).

For further investigation, we fixed the variable of surface chemistry and altered the dispersity. We dispersed these IO nanoparticles in phosphate buffered saline (PBS) to make them aggregated. It was as expected that nanoparticles were precipitated remarkably on the surface of cells, whether pipetting or not (Supplementary Figs S15, S16), followed by notable up-regulation of LC3-II (Fig. 3e,f). For IO@DMSA nanoparticles, who have better dispersity, also aggregated when dispersed in PBS and mixed by no pipetting, followed by increase of autophagic vacuoles (Supplementary Fig. S17). These distinct results from nanoparticles with the same surface coating strongly highlight the key role of the dispersity in autophagic effect. Surface ligands can modulate autophagic effect by improving the dispersity of nanoparticles. Once nanoparticles aggregate and precipitate, autophagic effect consequently happens regardless of surface ligands.

Size is also an important factor in cellular response towards nanoparticles^31^,^32^. Besides 27 nm IO particles, we investigated 10 nm and 100 nm IO particles. Particles larger than 100 nm are difficult to avoid sedimentation owning to weaker Brownian motion and stronger gravity^33^. As expected, 10 nm and 100 nm IO nanoparticles elicited autophagic effect when they were aggregated and precipitated. However, pipetting or protein adsorption promoted the dispersity of 10 nm and 100 nm IO nanoparticles and then diminished autophagic effect (Supplementary Figs S18, S19). Overall, IO particles with nano size elicited autophagic effect in a dispersity-dependent manner. We could modulate autophagy by IO nanoparticles through tuning their dispersity.

**Figure 4:**
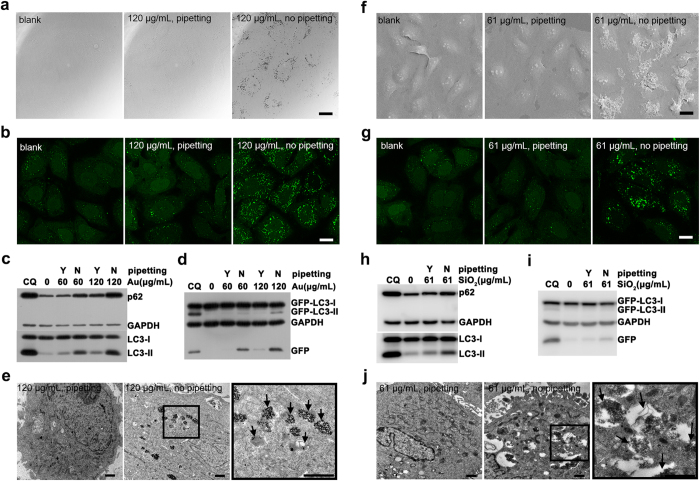
Modulating autophagy by gold and silica nanoparticles. **(a**–**e**) Cells were treated with Au@DMSA nanoparticles. (**a**) Confocal bright field images. (**b**) Fluorescence images of GFP-LC3. (**c**,**d**) Western blot analysis of autophagy-related proteins. (**e**) TEM images of cells. The black arrows in the right views pointed to autophagic vacuoles. (**f**–**j**) Cells were treated with SiO_2_-COOH nanoparticles. (**f**) SEM images of cells. (**g**) Confocal fluorescence images of GFP-LC3. (**h,i**) Western blot analysis of autophagy-related proteins. (**j**) TEM images of cells. The black arrows in the right views pointed to autophagic vacuoles. Cells were treated for 24 h. Scale bars were 20 μm (**a**,**b**,**f**,**g**) and 1 μm (**e**,**j**) respectively.

Besides IO nanoparticles, gold and silica nanoparticles have been extensively studied in nanobiotechnology and become promising nanomedical platforms for their excellent performance and biocompatibility^34^,^35^. Gold nanoparticles based CYT-6091^36^ and silica nanoparticles based Cornell Dots^37^ have been approved by FDA for clinical trial. Nonetheless, recent works reported gold and silica nanoparticles triggered autophagy^38^,^39^,^40^. We evaluated whether the autophagic effect mediated by these biocompatible nanoparticles was related to their dispersity. We coated 12 nm gold nanoparticles (Supplementary Fig. S20) with DMSA or sodium 2,3-dimercapto-1-propanesulfonate (DMPS) (Supplementary Fig. S21). Once dispersed in complete medium by no pipetting, Au@DMSA or Au@DMPS nanoparticles would aggregate and precipitate on the surface of cells, bringing on a large number of GFP-LC3 puncta (Fig. 4a,b and Supplementary Fig. S22). Western blot further confirmed the up-regulation of autophagy-related proteins (Fig. 4c,d and Supplementary Fig. S22). TEM demonstrated the accumulation of autophagic vacuoles (Fig. 4e). However, pipetting improved the dispersity of gold nanoparticles and diminished autophagic effect. The results showed that we could modulate autophagic effect induced by gold nanoparticles through tuning their dispersity.

We coated 28 nm silica nanoparticles with carboxylic acid (SiO_2_-COOH, ζ = −35.6 mV, pH 6.88) or quaternary ammonium cation (SiO_2_-QAC, ζ = +24.5 mV, pH 6.45). They showed considerable autophagic effect once aggregated and precipitated (Fig. 4f–j and Supplementary Fig. S23). While pipetting prevented nanoparticles from aggregating and eventually declined the autophagic effect. This indicated that we could modulate autophagic effect induced by silica nanoparticles through tuning their dispersity as well.

**Figure 5:**
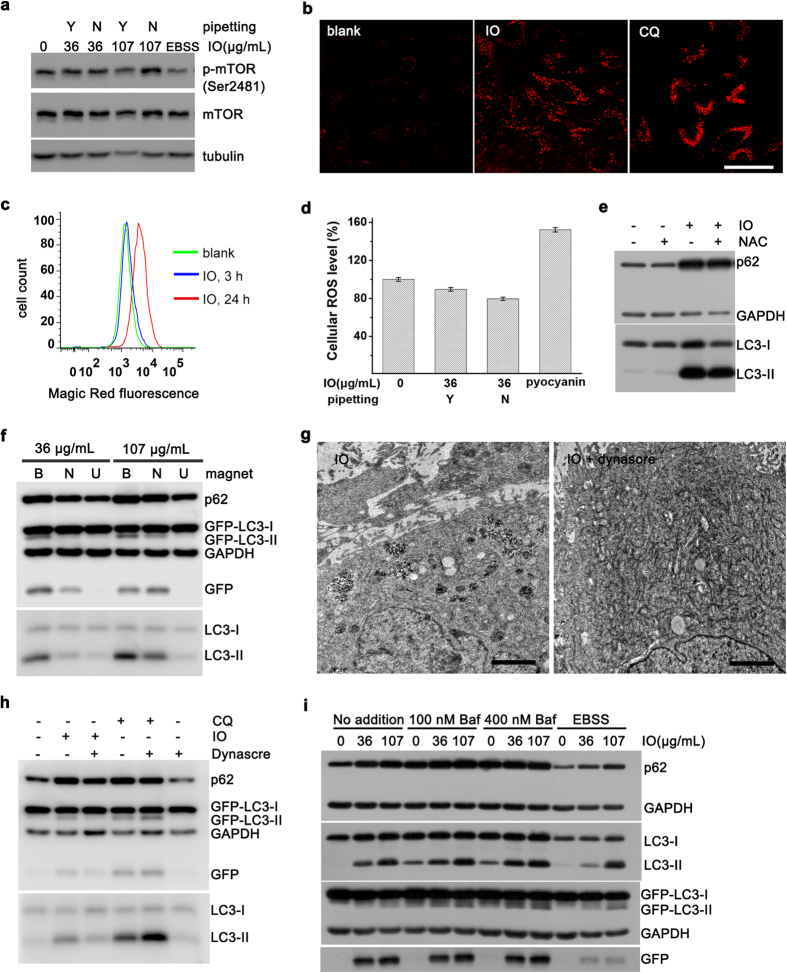
Concurrent cellular responses and autophagic flux assay. (**a**) Detection of phosphorylated mTOR. EBSS: When treated with EBSS only for 4 h, cells were starved so that mTOR was dephosphorylated and activated. (**b**) Immunofluorescence staining of LAMP1. Cells were treated with aggregated nanoparticles or CQ. Scale bar was 50 μm. (**c**) Enzyme activity of cathepsin B detected by Magic Red staining. (**d**) Cellular ROS analysis (*n* = 5. Data represent mean ± s.d.). Pyocyanin is an inducer of ROS. (**e**) Cells were treated with aggregated nanoparticles alone, or together with NAC. (**f**) Magnetic fields altered autophagy effect. B: below cells; N: no magnet; U: up cells. (**g**) TEM images, and (**h**) Western blot analysis of cells treated with aggregated nanoparticles alone, or together with dynasore for 12 h. Scale bar was 2 μm. (**i**) Cells were treated with aggregated nanoparticles alone or together with Bafilomycin A1 (Baf) or EBSS at final 5 h. Cells were treated for 24 h unless noted otherwise.

We next studied the possible mechanism of dispersity-dependent autophagic effect. Mammalian target of rapamycin (mTOR) is a major regulator of autophagy. When dephosphorylated, mTOR will be inhibited and then trigger autophagy. Starvation (*e.g.*, treating cells with Earle’s Balanced Salt Solution, EBSS) is an effective method to inhibit mTOR^19^. We found that mTOR was not dephosphorylated and not inhibited by IO@citrate nanoparticles (Fig. 5a), indicating an mTOR-independent manner. Lysosomal dysfunction and oxidative stress are two major mechanisms by which nanoparticles trigger autophagy^12^. Nonetheless, lysosome or autolysosome increased after treated with aggregated nanoparticles (Figs 1d and 5b), followed by enhanced total activity of lysosomal enzyme (Fig. 5c). We did not observe any elevation of cellular reactive oxygen species (ROS) (Fig. 5d and Supplementary Fig. S24). N-acetyl-cysteine (NAC), a ROS scavenger, failed to decline autophagic effect (Fig. 5e), indicating a non-essential role of oxidative stress in autophagic effect. Additionally, the vitality of cells treated with aggregated nanoparticles remained about 90% or even higher (Supplementary Fig. S24), strongly supporting the excellent biocompatibility of IO nanoparticles. As aggregated and precipitated nanoparticles will adhere to cell surface and cause a concomitant increase of endocytosis^41^, uptake of nanoparticles may be a key factor in autophagic effect. We put an NdFeB magnet up cells to reduce adhesion of nanoparticles. Subsequently, we observed the decrease in uptake and autophagic effect. Conversely, a magnet below cells would enhance adhesion, increase uptake and promote autophagic effect (Fig. 5f and Supplementary Fig. S25). We further blocked the uptake of nanoparticles by dynasore (Fig. 5g), an inhibitor of dynamin^42^. As a result, autophagic effect was dramatically diminished; meanwhile dynasore itself did not block autophagy (Fig. 5h). These results strongly supported that the distinct endocytosis amount of nanoparticles may account for the dispersity-dependent autophagic effect.

To explore how these internalized nanoparticles induced autophagic effect, we further studied the autophagy flux using bafilomycin A1 and EBSS^19^. We treated cells with aggregated nanoparticles for 19 hours, and then engaged bafilomycin A1 to block autophagy flux, or treated cells with EBSS to accelerate autophagy flux for 5 hours. There was only a little increase of autophagosome in the presence of bafilomycin A1 (Fig. 5i). This may be due to the nearly saturated accumulation of autophagosomes. GFP fragment as a partial degradation product showed the moderate activity of lysosomes. As expected, once activated lysosome by EBSS, the residual GFP fragment was degraded rapidly (Fig. 5i). These results suggested that the increase of autophagic vacuoles may due to the enhancement of autophagy induction and the unchanged rate of degradation in autolysosomes.

**Figure 6:**
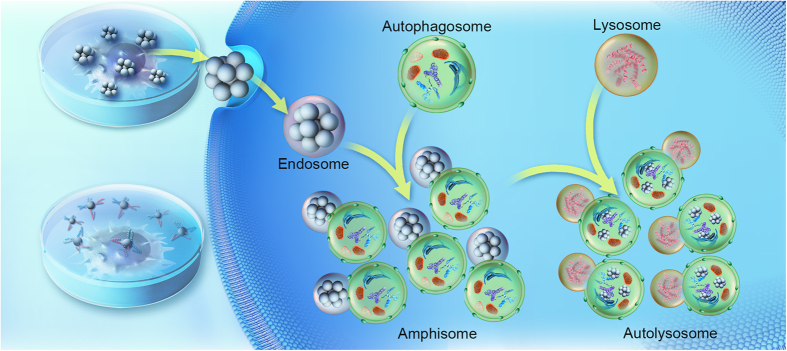
A probable process that aggregated nanoparticles induce autophagic effect. Aggregated nanoparticles enter into cells largely and are regarded as autophagic cargos. The as- formed endosomes initialize autophagy or fuse with autophagosomes to form amphisomes. Excess cargos saturate autophagy capacity and cause accumulation of amphisomes. The activity of lysosomes do not increase after fuse with amphisomes. The degradation of cargos in autolysosomes is slow due to the existence of highly stable nanoparticles; as a result, the number of amphisomes and autolysosomes increase dramatically inside cells. This figure was designed and drawn by Dengtong Huang.

Based on the results obtained, we propose a reasonable cellular process as follows (Fig. 6). Aggregated nanoparticles enter into cells by endocytosis much more than well-dispersed nanoparticles. The internalized nanoparticles may be regarded as foreign materials and autophagic cargos by cells and then trigger autophagy. As a result, the as-formed endosomes initialize the formation of autophagosomes or fuse with mature autophagosomes to form amphisomes. However, a large increase of autophagic cargos saturates the autophagy capacity. Autophagosomes or amphisomes significantly increase. The activity of lysosomes is not further increasing after fusing with autophagosomes, leading to accumulation of autolysosomes. Alternatively, these autophagic substrate proteins are degraded slowly (Supplementary Fig. S6) due to the existence of highly stable inorganic nanoparticles, which are difficult to be digested even in activated lysosomes. In brief, aggregated nanoparticles supply more autophagic cargos while cannot accelerate the degradation of cargos in autolysosomes, resulting in high accumulation of autophagic vacuoles.

## Discussion

We have demonstrated that nanoparticles induce autophagy in a dispersity-dependent manner and we could modulate autophagy through tuning the dispersity of nanoparticles. Several univariate experiments comprehensively reveal the key role of the dispersity in autophagic effect mediated by nanoparticles. IO nanoparticles strongly elicited autophagic effect due to their aggregation. When other variables were fixed, pipetting, dilution, BSA adsorption and surface modification improved the dispersity of nanoparticles and therefore declined autophagic effect. Furthermore, we also demonstrate that autophagic effect mediated by gold nanoparticles and silica nanoparticles is likewise dependent on their dispersity. This dispersity-dependent autophagic effect is probably a universal cellular response to nanoparticles.

Our results suggest that the endocytosis of nanoparticles is responsible for the dispersity-dependent autophagy. Nevertheless, why aggregated nanoparticles enter into cells more easily remains unclear. Of course, it could be accepted that aggregated nanoparticles sediment quickly and adhere to the surface of cells, followed by the uptake of nanoparticles. However, does cells internalize all objects adhering to their surface? Another derived question is that how these internalized nanomaterials induce autophagy. Cells internalize proteins and other nutrition from time to time. Why these internalized cargos do not induce autophagy? We may quickly provide an answer that nutrition can be digested and utilized by cells while nanomaterials cannot, especially those inorganic materials. It seems quite right because we observed much autophagic vacuoles containing nanoparticles inside cells. Nonetheless, we do not known the ultimate fate of the internalized nanomaterials for long term. All these questions are very important for particle-cell interaction, as well as the cell research. We are also on the way to the answer.

Although we do not completely clarify the detail mechanism, our finding suggests a new way for modulation of autophagy by tuning the dispersity of nanoparticles, which may provide a new strategy in cancer therapy. The role of autophagy in cancer is complex and likely dependent on tumor context^43^. Tunable autophagic effect shows great advantage when targeting autophagy for cancer therapy. In cases where autophagy compromises the efficacy of cancer therapy, inhibition of autophagy can be a suitable therapeutic strategy^44^,^45^,^46^,^47^. pH-triggered aggregated nanoparticles could selectively aggregate in tumor microenvironment^48^ and fill up cancer cells with nanoparticles-containing autophagosomes. As a result, the autophagic degradation of damaged molecules caused by chemotherapy or radiotherapy is blocked, which may sensitize cancer cells to chemotherapy and radiotherapy. In cases where autophagy is defective, we need nanoparticles with better dispersity to avoid aggregation.

In summary, we have demonstrated that autophagic effect mediated by biocompatible nanoparticles is mainly dependent on their dispersity. Dispersity-dependent autophagic effect is probably a common feature shared by most types of nanoparticles and offers a new strategy for cancer therapy by altering the dispersity of nanoparticles. This phenomenon likely results from high uptake and accumulation of aggregated nanoparticles. Furthermore, dispersity-dependent autophagic effect suggests that tuning the dispersity of nanoparticles can be a new way to modulate autophagy process.

## Methods

### Pipetting or not

In a typical procedure of pipetting, added 990 μL of complete medium to 10 μL of IO@citrate nanoparticles and then mixed by pipetting immediately. The resulted nanoparticles were well-dispersed. There was no big difference between pipetting for 10 seconds and 60 seconds. In a procedure of no pipetting, added 10 μL of IO@citrate nanoparticles to 990 μL of complete medium and then shook gently. The resulted nanoparticles were aggregated. There was no notable difference when gently shook after one minute.

### Dilution

Added 10 μL of IO@citrate nanoparticles to each of 10 tubes and then supplied 0, 10, 20, 30, 40, 50, 60, 70, 80, 90 μL of ultrapure water (18.2 MΩ.cm, Purelab flex 3, ELGA). Mixed the diluted nanoparticles with complete medium by no pipetting. Afterwards, supplemented 90, 80, 70, 60, 50, 40, 30, 20, 10, 0 μL of pure water, respectively, to eliminate excess variables.

### BSA adsorption

Mixed 4 μL of 0, 0.5, 2, 10, 40 mg/mL BSA (fraction V, cold alcohol isolation, BBI) aqueous solution with 36 μL of IO@citrate nanoparticles. Put 30 μL of mixture solution to complete medium by no pipetting. Afterwards, supplemented BSA aqueous and pure water to make the concentrations of BSA and nanoparticles equal in each group.

### Comparison of IO nanoparticles with different coating

For comparison of IO@citrate, IO@DA and IO@DOPAC nanoparticles, their concentrations were adjusted to the same value. They were all added at the same initial concentration and the same final concentration. The same did the comparison of IO@citrate and IO@DMSA. Feraheme (ferumoxytol, AMAG) was added at much higher initial concentration (15 mg/mL) and final concentration (up to 300 μg/mL) than IO@citrate. Feraheme is highly stable even dispersed in 10x PBS.

### Aggregation in PBS

For IO nanoparticles (@DA, @DOPAC, @DMDA) which had better stability in saline than IO@citrate nanoparticles, we dispersed them in PBS to make them aggregated. In a typical procedure, 90 μL of IO@DA nanoparticles was added into 10 μL of 10x PBS (dispersed in PBS) or pure water (dispersed in water), mixed for just seconds or several hours (IO@DMSA nanoparticles need more time to get aggregated). Then the mixture solution was added into complete medium by pipetting or not.

## Additional Information

**How to cite this article**: Huang, D. *et al.* Nanoparticles modulate autophagic effect in a dispersity-dependent manner. *Sci. Rep.* **5**, 14361; doi: 10.1038/srep14361 (2015).

## Supplementary Material

Supplementary Information

## References

[b1] Klionsky D. J. & Emr S. D. Autophagy as a regulated pathway of cellular degradation. Science 290, 1717–1721 (2000). 11099404 10.1126/science.290.5497.1717 PMC2732363

[b2] Xie Z. & Klionsky D. J. Autophagosome formation: Core machinery and adaptations. Nat. Cell Biol. 9, 1102–1109 (2007). 17909521 10.1038/ncb1007-1102

[b3] Mizushima N. & Komatsu M. Autophagy: Renovation of cells and tissues. Cell 147, 728–741 (2011). 22078875 10.1016/j.cell.2011.10.026

[b4] Murrow L. & Debnath J. Autophagy as a stress-response and quality-control mechanism: Implications for cell injury and human disease. Annu. Rev. Pathol-Mech. 8, 105–137 (2013). 10.1146/annurev-pathol-020712-163918 PMC3971121 23072311

[b5] Shintani T. & Klionsky D. J. Autophagy in health and disease: A double-edged sword. Science 306, 990–995 (2004). 15528435 10.1126/science.1099993 PMC1705980

[b6] Mizushima N., Levine B., Cuervo A. M. & Klionsky D. J. Autophagy fights disease through cellular self-digestion. Nature 451, 1069–1075 (2008). 18305538 10.1038/nature06639 PMC2670399

[b7] Rubinsztein D. C., Codogno P. & Levine B. Autophagy modulation as a potential therapeutic target for diverse diseases. Nat. Rev. Drug Discov. 11, 709–730 (2012). 22935804 10.1038/nrd3802 PMC3518431

[b8] Vakifahmetoglu-Norberg H., Xia H. G. & Yuan J. Pharmacologic agents targeting autophagy. J. Clin. Invest. 125, 5–13 (2015). 25654545 10.1172/JCI73937 PMC4382252

[b9] Baek K.-H., Park J. & Shin I. Autophagy-regulating small molecules and their therapeutic applications. Chem. Soc. Rev. 41, 3245–3263 (2012). 22293658 10.1039/c2cs15328a

[b10] Tong X. P. *et al.* Key autophagic targets and relevant small-molecule compounds in cancer therapy. Cell Proliferation 48, 7–16 (2015). 25474301 10.1111/cpr.12154 PMC6496147

[b11] Stern S., Adiseshaiah P. & Crist R. Autophagy and lysosomal dysfunction as emerging mechanisms of nanomaterial toxicity. Part. Fibre. Toxicol. 9, 20 (2012). 22697169 10.1186/1743-8977-9-20 PMC3441384

[b12] Peynshaert K. *et al.* Exploiting intrinsic nanoparticle toxicity: The pros and cons of nanoparticle-induced autophagy in biomedical research. Chem. Rev. 114, 7581–7609 (2014). 24927160 10.1021/cr400372p

[b13] Man N., Yu S.-H. & Wen L.-P. Rare earth oxide nanocrystals as a new class of autophagy inducers. Autophagy 6, 310–311 (2010). 20104026 10.4161/auto.6.2.11138

[b14] Zabirnyk O., Yezhelyev M. & Seleverstov O. Nanoparticles as a novel class of autophagy activators. Autophagy 3, 278–281 (2007). 17351332 10.4161/auto.3916

[b15] Zhang Q. *et al.* Autophagy-mediated chemosensitization in cancer cells by fullerene C60 nanocrystal. Autophagy 5, 1107–1117 (2009). 19786831 10.4161/auto.5.8.9842

[b16] Lu Y. *et al.* MnO nanocrystals: A platform for integration of MRI and genuine autophagy induction for chemotherapy. Adv. Funct. Mater. 23, 1534–1546 (2013).

[b17] Laurent S. *et al.* Magnetic iron oxide nanoparticles: Synthesis, stabilization, vectorization, physicochemical characterizations, and biological applications. Chem. Rev. 108, 2064–2110 (2008). 18543879 10.1021/cr068445e

[b18] Reddy L. H., Arias J. L., Nicolas J. & Couvreur P. Magnetic nanoparticles: Design and characterization, toxicity and biocompatibility, pharmaceutical and biomedical applications. Chem. Rev. 112, 5818–5878 (2012). 23043508 10.1021/cr300068p

[b19] Klionsky D. J. *et al.* Guidelines for the use and interpretation of assays for monitoring autophagy. Autophagy 8, 445–544 (2012). 22966490 10.4161/auto.19496 PMC3404883

[b20] Pankiv S. *et al.* p62/SQSTM1 binds directly to Atg8/LC3 to facilitate degradation of ubiquitinated protein aggregates by autophagy. J. Biol. Chem. 282, 24131–24145 (2007). 17580304 10.1074/jbc.M702824200

[b21] Kabeya Y. *et al.* LC3, a mammalian homologue of yeast Apg8p, is localized in autophagosome membranes after processing. EMBO J. 19, 5720–5728 (2000). 11060023 10.1093/emboj/19.21.5720 PMC305793

[b22] Mizushima N., Yamamoto A., Matsui M., Yoshimori T. & Ohsumi Y. *In vivo* analysis of autophagy in response to nutrient starvation using transgenic mice expressing a fluorescent autophagosome marker. Mol. Biol. Cell 15, 1101–1111 (2004). 14699058 10.1091/mbc.E03-09-0704 PMC363084

[b23] Ichimura Y., Kominami E., Tanaka K. & Komatsu M. Selective turnover of p62/A170/SQSTM1 by autophagy. Autophagy 4, 1063–1066 (2008). 18776737 10.4161/auto.6826

[b24] Ni H.-M. *et al.* Dissecting the dynamic turnover of GFP-LC3 in the autolysosome. Autophagy 7, 188–204 (2011). 21107021 10.4161/auto.7.2.14181 PMC3039769

[b25] Eskelinen E.-L. Roles of LAMP-1 and LAMP-2 in lysosome biogenesis and autophagy. Mol. Aspects Med. 27, 495–502 (2006). 16973206 10.1016/j.mam.2006.08.005

[b26] Chan L. L.-Y. *et al.* A novel image-based cytometry method for autophagy detection in living cells. Autophagy 8, 1371–1382 (2012). 22895056 10.4161/auto.21028 PMC3442883

[b27] Wells M. A., Abid A., Kennedy I. M. & Barakat A. I. Serum proteins prevent aggregation of Fe2O3 and ZnO nanoparticles. Nanotoxicology 6, 837–846 (2012). 22149273 10.3109/17435390.2011.625131 PMC3963816

[b28] Dominguez-Medina S., Blankenburg J., Olson J., Landes C. F. & Link S. Adsorption of a protein monolayer via hydrophobic interactions prevents nanoparticle aggregation under harsh environmental conditions. ACS Sustain. Chem. Eng. 1, 833–842 (2013). 23914342 10.1021/sc400042h PMC3731158

[b29] Zhang Y. *et al.* Tuning the autophagy-inducing activity of lanthanide-based nanocrystals through specific surface-coating peptides. Nat. Mater. 11, 817–826 (2012). 22797828 10.1038/nmat3363

[b30] Wu L. *et al.* Tuning cell autophagy by diversifying carbon nanotube surface chemistry. ACS Nano 8, 2087–2099 (2014). 24552177 10.1021/nn500376w PMC5586106

[b31] Jiang W., Kim B. Y. S., Rutka J. T. & Chan W. C. W. Nanoparticle-mediated cellular response is size-dependent. Nat. Nanotechnol. 3, 145–150 (2008). 18654486 10.1038/nnano.2008.30

[b32] Albanese A., Tang P. S. & Chan W. C. W. The effect of nanoparticle size, shape, and surface chemistry on biological systems. Annu. Rev. Biomed. Eng. 14, 1–16 (2012). 22524388 10.1146/annurev-bioeng-071811-150124

[b33] Cho E. C., Zhang Q. & Xia Y. The effect of sedimentation and diffusion on cellular uptake of gold nanoparticles. Nat. Nanotechnol. 6, 385–391 (2011). 21516092 10.1038/nnano.2011.58 PMC3227810

[b34] Boisselier E. & Astruc D. Gold nanoparticles in nanomedicine: Preparations, imaging, diagnostics, therapies and toxicity. Chem. Soc. Rev. 38, 1759–1782 (2009). 19587967 10.1039/b806051g

[b35] Lee J. E., Lee N., Kim T., Kim J. & Hyeon T. Multifunctional mesoporous silica nanocomposite nanoparticles for theranostic applications. Acc. Chem. Res. 44, 893–902 (2011). 21848274 10.1021/ar2000259

[b36] Libutti S. K. *et al.* Phase I and pharmacokinetic studies of CYT-6091, a novel PEGylated colloidal gold-rhTNF nanomedicine. Clin. Cancer. Res. 16, 6139–6149 (2010). 20876255 10.1158/1078-0432.CCR-10-0978 PMC3004980

[b37] Benezra M. *et al.* Multimodal silica nanoparticles are effective cancer-targeted probes in a model of human melanoma. J. Clin. Invest. 121, 2768–2780 (2011). 21670497 10.1172/JCI45600 PMC3223837

[b38] Ma X. *et al.* Gold nanoparticles induce autophagosome accumulation through size-dependent nanoparticle uptake and lysosome impairment. ACS Nano 5, 8629–8639 (2011). 21974862 10.1021/nn202155y

[b39] Ha S.-W., Weitzmann M. N. & Beck G. R. Bioactive silica nanoparticles promote osteoblast differentiation through stimulation of autophagy and direct association with LC3 and p62. ACS Nano 8, 5898–5910 (2014). 24806912 10.1021/nn5009879 PMC4076025

[b40] Duan J. *et al.* Silica nanoparticles enhance autophagic activity, disturb endothelial cell homeostasis and impair angiogenesis. Part. Fibre. Toxicol. 11, 50 (2014). 25266717 10.1186/s12989-014-0050-8 PMC4193984

[b41] Lesniak A. *et al.* Nanoparticle adhesion to the cell membrane and its effect on nanoparticle uptake efficiency. J. Am. Chem. Soc. 135, 1438–1444 (2013). 23301582 10.1021/ja309812z

[b42] Macia E. *et al.* Dynasore, a cell-permeable inhibitor of dynamin. Dev. Cell 10, 839–850 (2006). 16740485 10.1016/j.devcel.2006.04.002

[b43] Kimmelman A. C. The dynamic nature of autophagy in cancer. Genes Dev. 25, 1999–2010 (2011). 21979913 10.1101/gad.17558811 PMC3197199

[b44] Apel A., Herr I., Schwarz H., Rodemann H. P. & Mayer A. Blocked autophagy sensitizes resistant carcinoma cells to radiation therapy. Cancer Res. 68, 1485–1494 (2008). 18316613 10.1158/0008-5472.CAN-07-0562

[b45] Lamoureux F. *et al.* Blocked autophagy using lysosomotropic agents sensitizes resistant prostate tumor cells to the novel Akt inhibitor AZD5363. Clin. Cancer. Res. 19, 833–844 (2013). 23258740 10.1158/1078-0432.CCR-12-3114

[b46] Sui X. *et al.* Autophagy and chemotherapy resistance: A promising therapeutic target for cancer treatment. Cell Death Dis. 4, e838 (2013). 24113172 10.1038/cddis.2013.350 PMC3824660

[b47] White E. The role for autophagy in cancer. J. Clin. Invest. 125, 42–46 (2015). 25654549 10.1172/JCI73941 PMC4382247

[b48] Li H. *et al.* “Mixed-charge self-assembled monolayers” as a facile method to design pH-induced aggregation of large gold nanoparticles for near-infrared photothermal cancer therapy. ACS. Appl. Mater. Inter. 6, 18930–18937 (2014). 10.1021/am504813f 25286378

