# Supplementary material for: Nanoparticles modulate autophagic effect in a dispersity-dependent manner
Source: Sci Rep. 2015 Sep 23;5:14361. doi: 10.1038/srep14361 (PMC4585824; doi:10.1038/srep14361)
Supplement: Supplementary Information [file srep14361-s1.pdf]

Nanoparticles modulate autophagic effect in a dispersity-dependent manner

Dengtong Huang, Hualu Zhou, and Jinhao Gao*

The Key Laboratory for Chemical Biology of Fujian Province, State Key Laboratory of Physical Chemistry of Solid Surfaces, and Department of Chemical Biology, College of Chemistry and Chemical Engineering, Xiamen University, Xiamen 361005, China.

*Email: jhgao@xmu.edu.cn (J.G.)

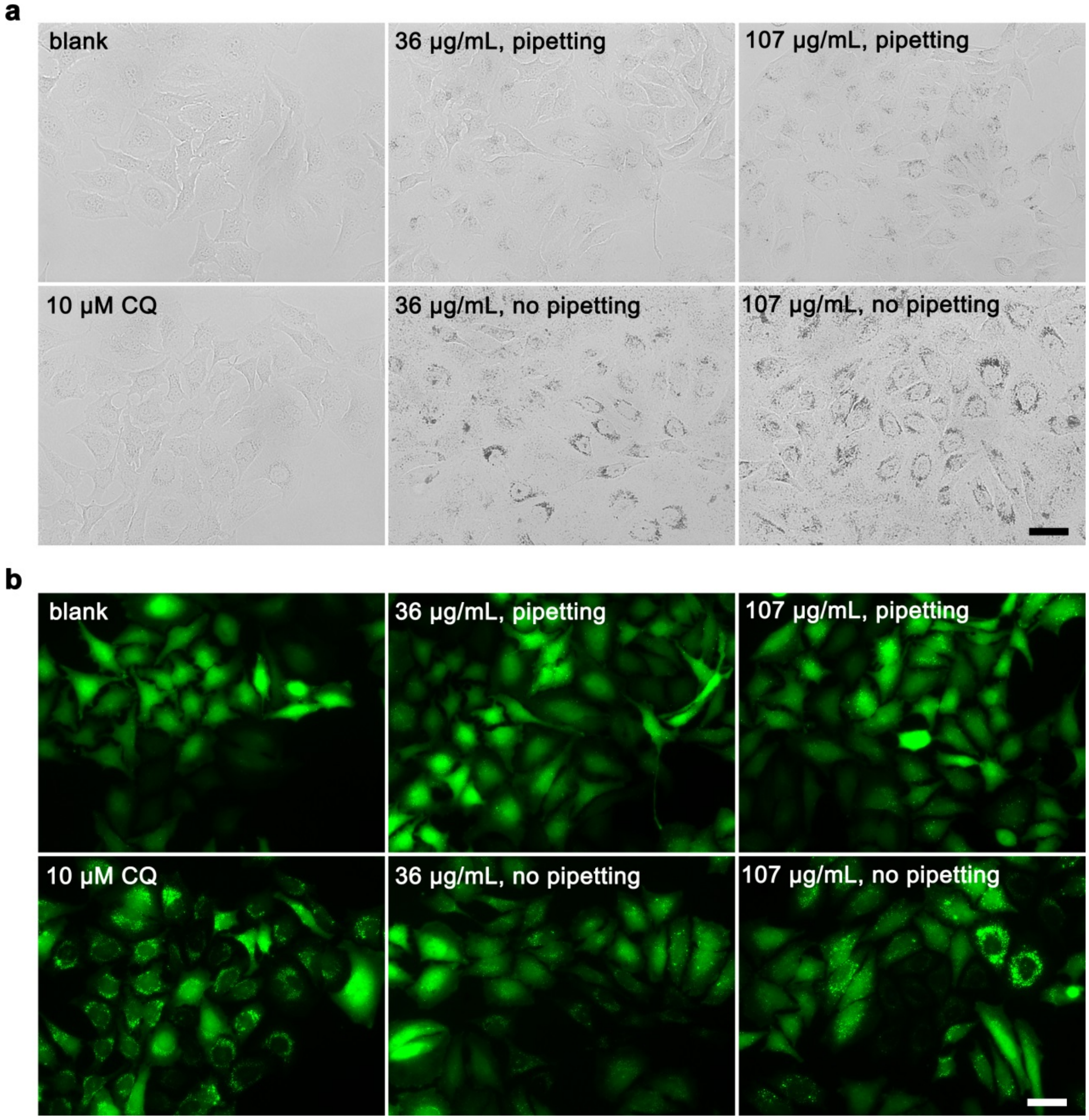

Supplementary Figure 1. IO@citrate nanoparticles aggregations induce accumulation of autophagic vacuoles. **a**, Bright field images and **b**, Fluorescence images of HeLa-GFP-LC3 cells after treatment for 24 h. Scale bars were 50 μm.

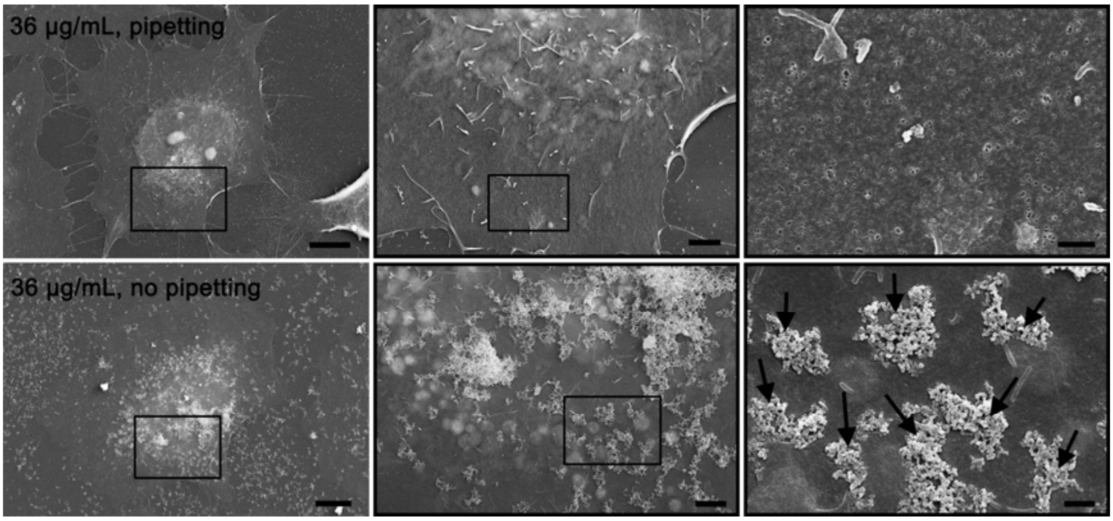

Supplementary Figure 2. Visualized adhesion of IO@citrate nanoparticles on the surface of cells by SEM. The views were magnified from left to right. Black arrows in the bottom right pointed to nanoparticle aggregations on the surface of cells. Scale bars were 10 μm (left), 2 μm (middle) and 0.5 μm (right) respectively.

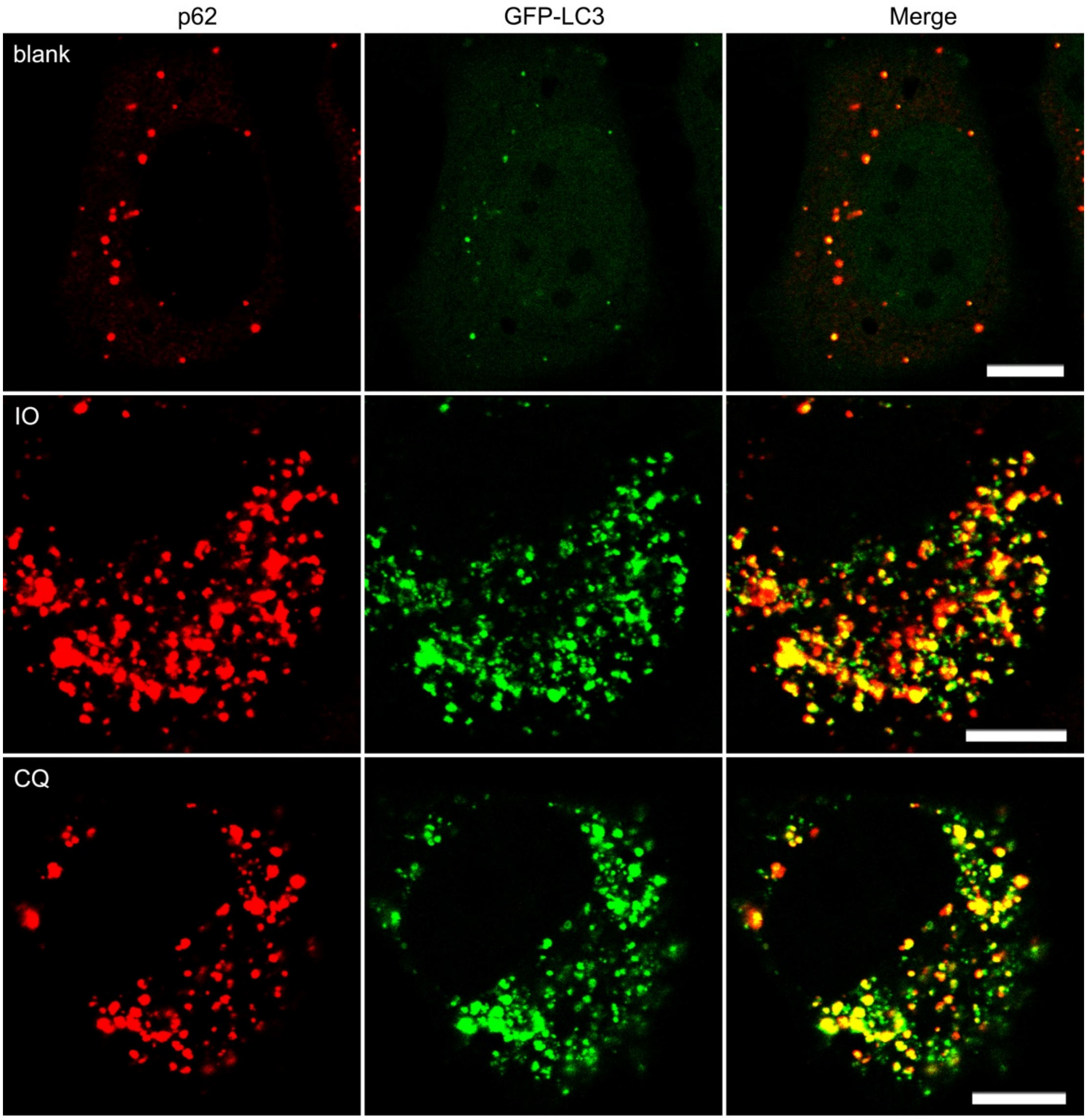

Supplementary Figure 3. Immunofluorescence colocation of GFP-LC3 puncta and p62 puncta. Cells were treated with IO@citrate nanoparticles by no pipetting or 50 μM CQ. Scale bars were 10 μm.

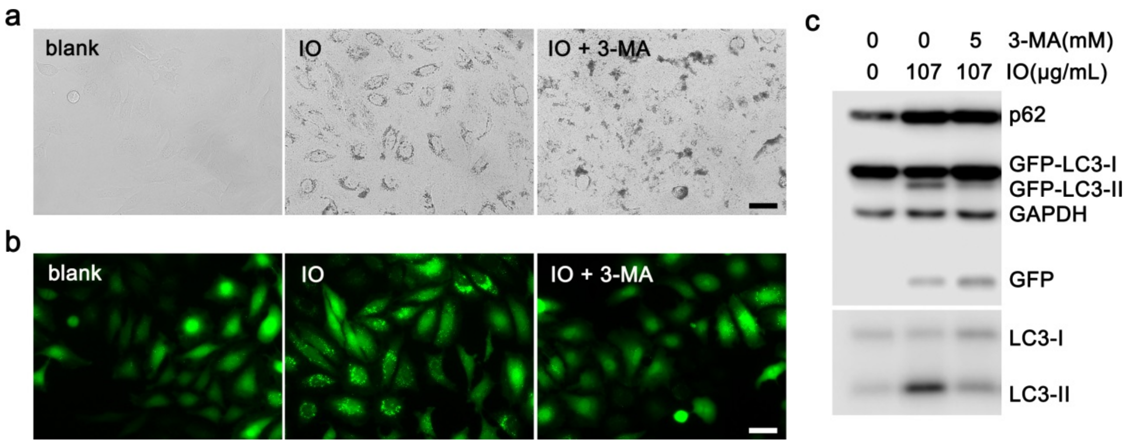

Supplementary Figure 4. 3-methyladenine (3-MA) inhibited autophagic effect induced by aggregated nanoparticles. Cells were treated with IO@citrate (no pipetting) alone or together with 3-MA for 15 h. **a**, Bright field images. **b**, Fluorescence images of GFP-LC3. **c**, Western blot analysis of autophagy-related proteins. Scale bars were 50 μm.

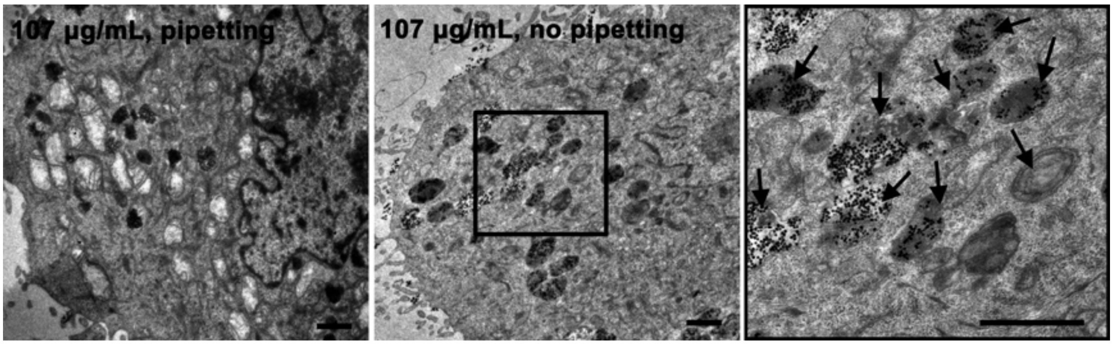

Supplementary Figure 5. TEM images of HeLa-GFP-LC3 cells after treatments. The right view was the magnification of the region with a black box in the middle view. The black arrows pointed to autophagic vacuoles. Scale bars were 1 μm.

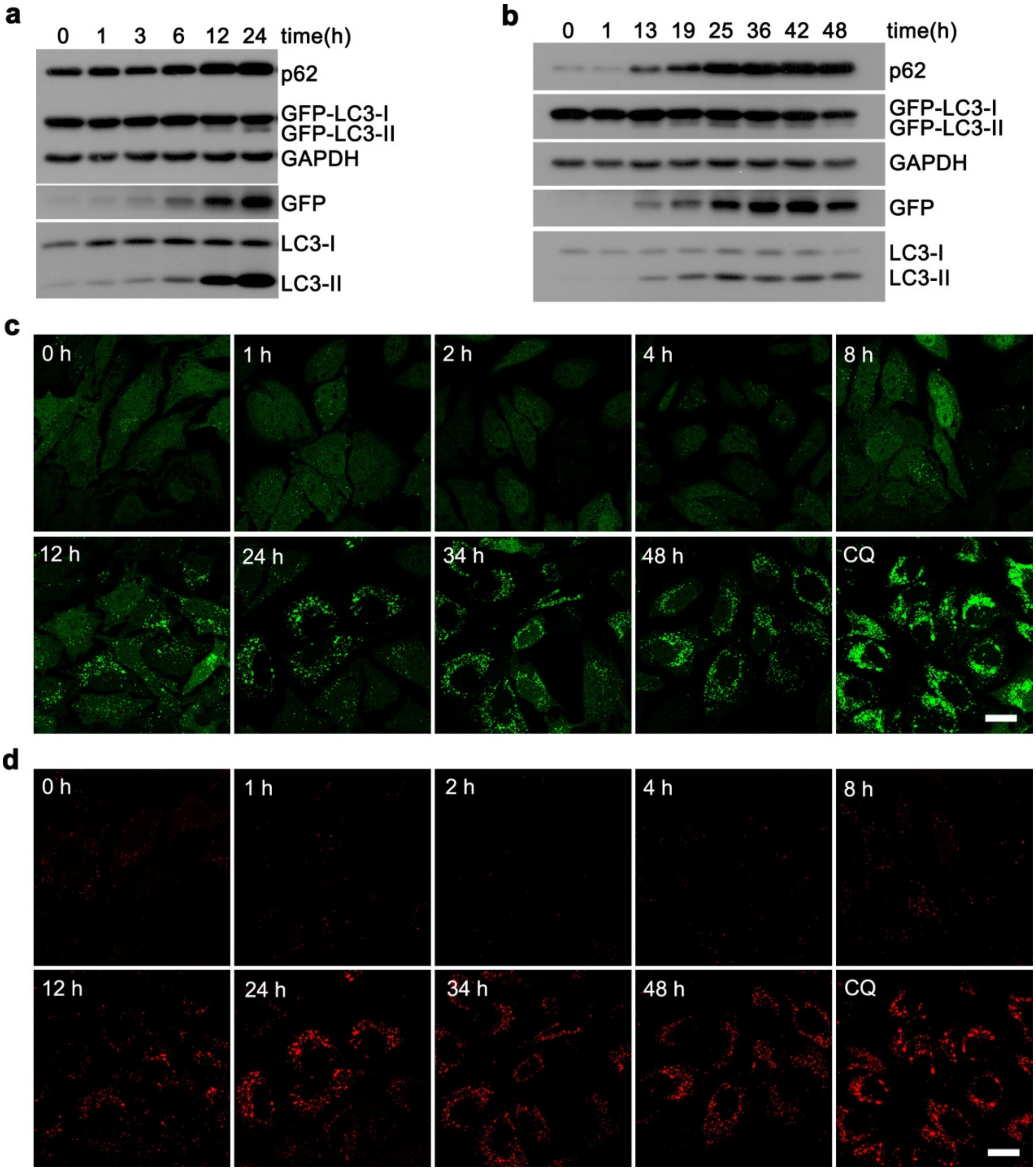

Supplementary Figure 6. Time-dependent accumulation of autophagic vacuoles. HeLa-GFP-LC3 cells were treated with aggregated IO@citrate nanoparticles (107 μg/mL) by no pipetting for different time. **a**-**b**, Western blot analysis of autophagy-related proteins. **c**, Confocal fluorescence images of GFP-LC3 puncta. **d**, Immunofluorescence images of LC3B. Scale bars were 25 μm.

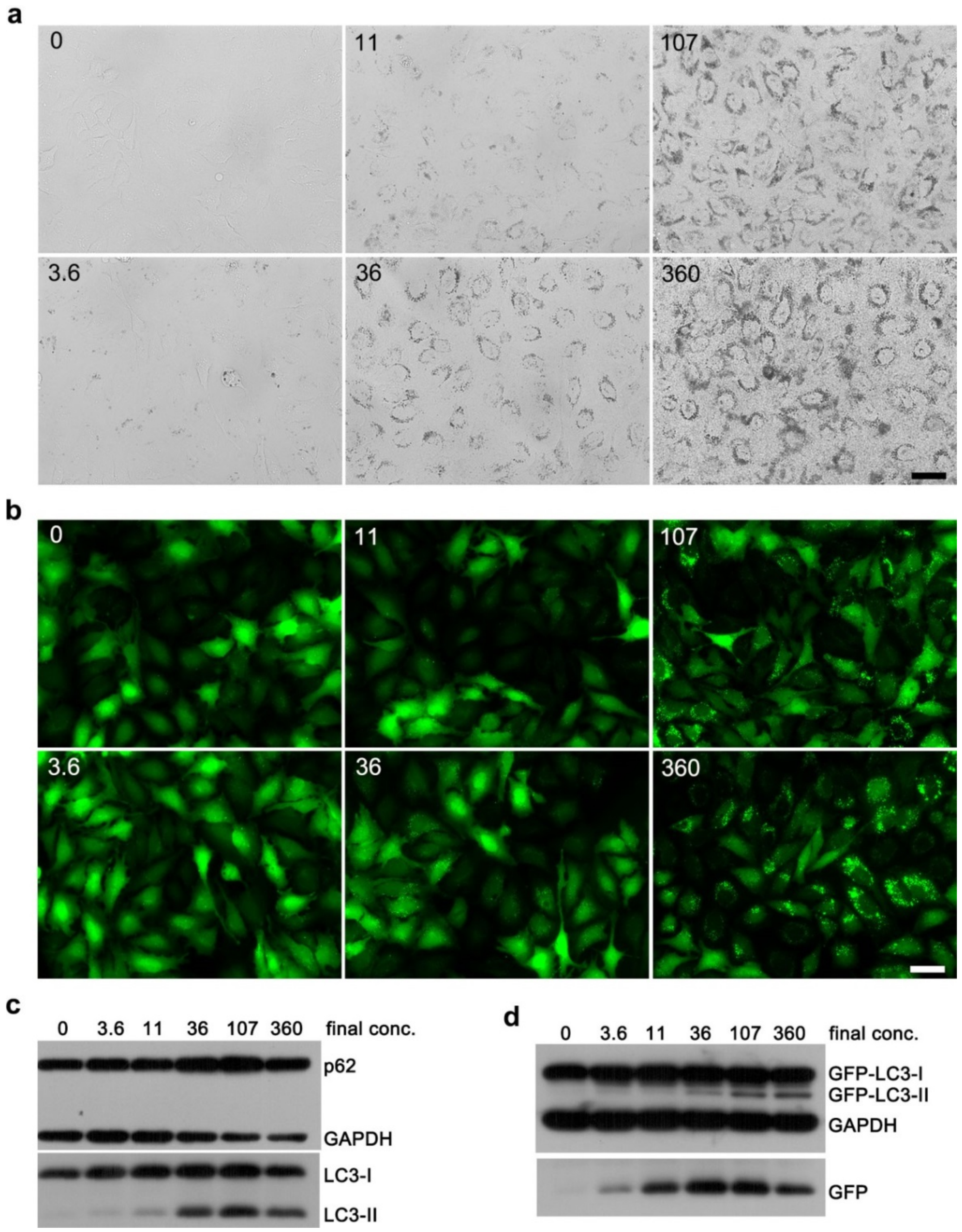

Supplementary Figure 7. Dose-dependent accumulation of autophagic vacuoles induced by IO@citrate nanoparticles. Cells were treated with various concentration (μg/mL) of aggregated nanoparticles for 24 h. **a**, Bright field images. **b**, Fluorescence images of GFP-LC3. **c-d**, Western blot analysis of autophagy-related proteins. Scale bars were 50 μm.

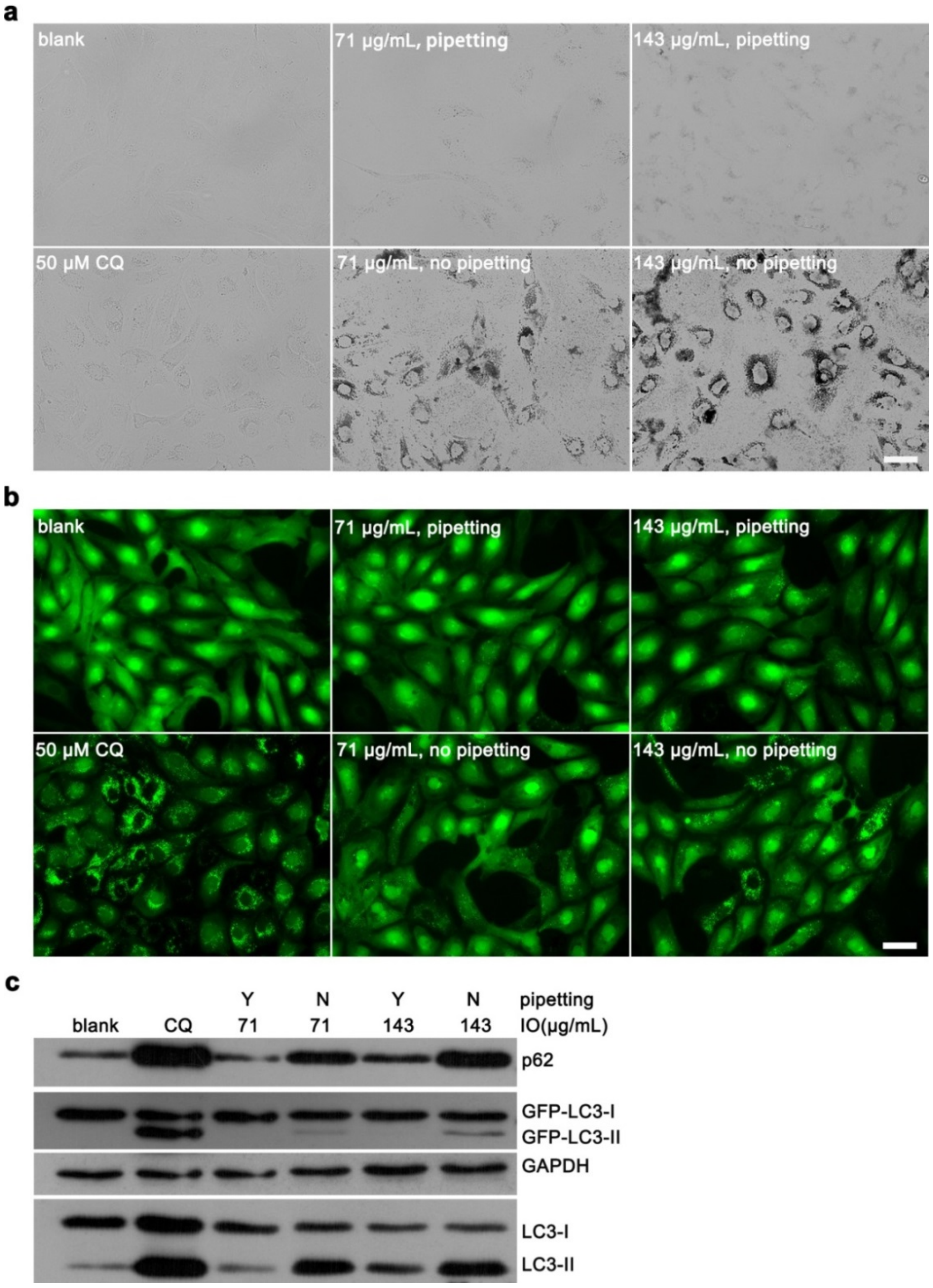

Supplementary Figure 8. IO@citrate nanoparticles induced accumulation of autophagic vacuoles by dispersity dependent in SKOV3-GFP-LC3 cells. **a**, Bright field images. **b**, Fluorescence images of GFP-LC3 puncta. **c**, Western blot analysis of autophagy-related proteins. Scale bars were 50 μm.

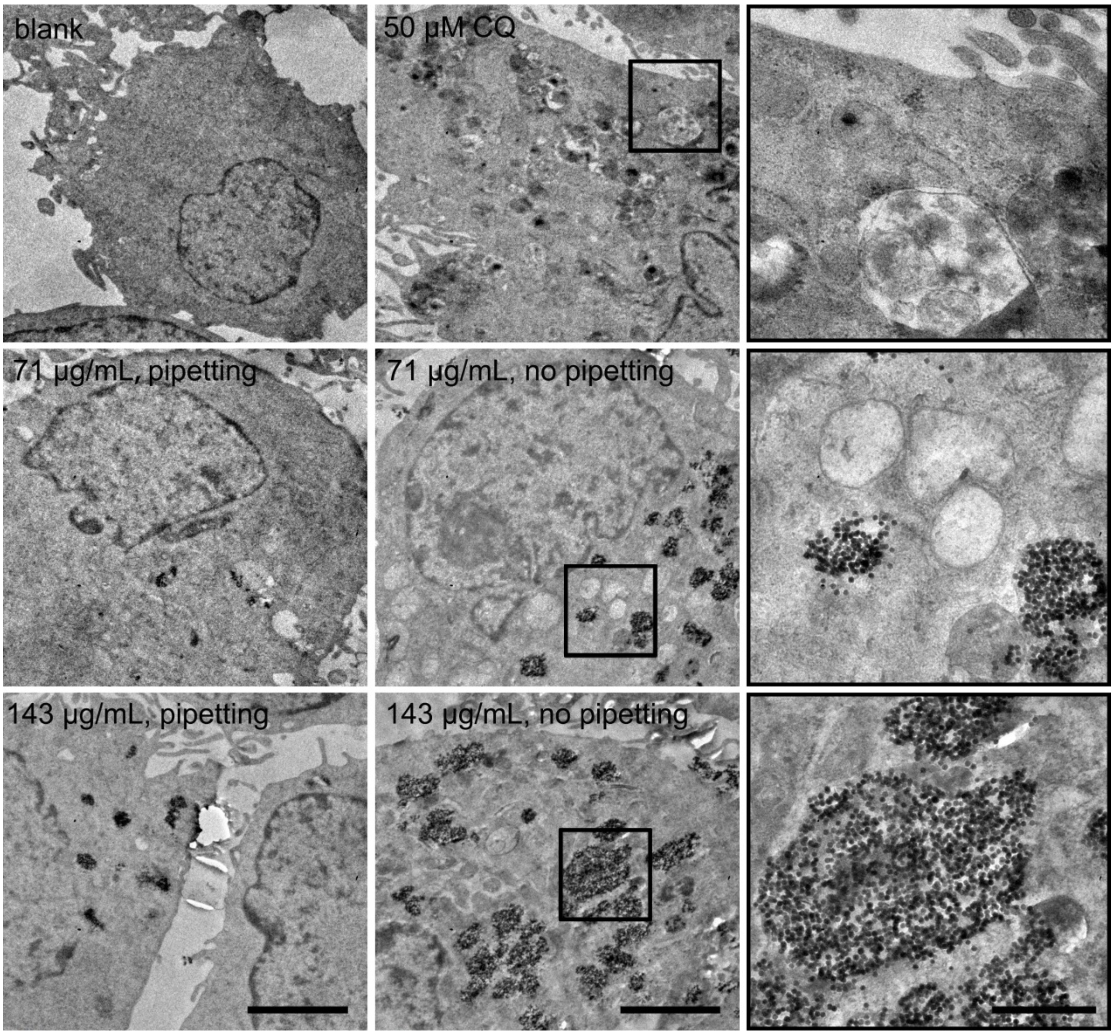

Supplementary Figure 9. TEM images of SKOV3-GFP-LC3 cells treated with IO@citrate nanoparticles. The right views were the magnification of the region with a black box in the middle views. Scale bars were 2 μm (left and middle), and 0.5 μm (right) respectively.

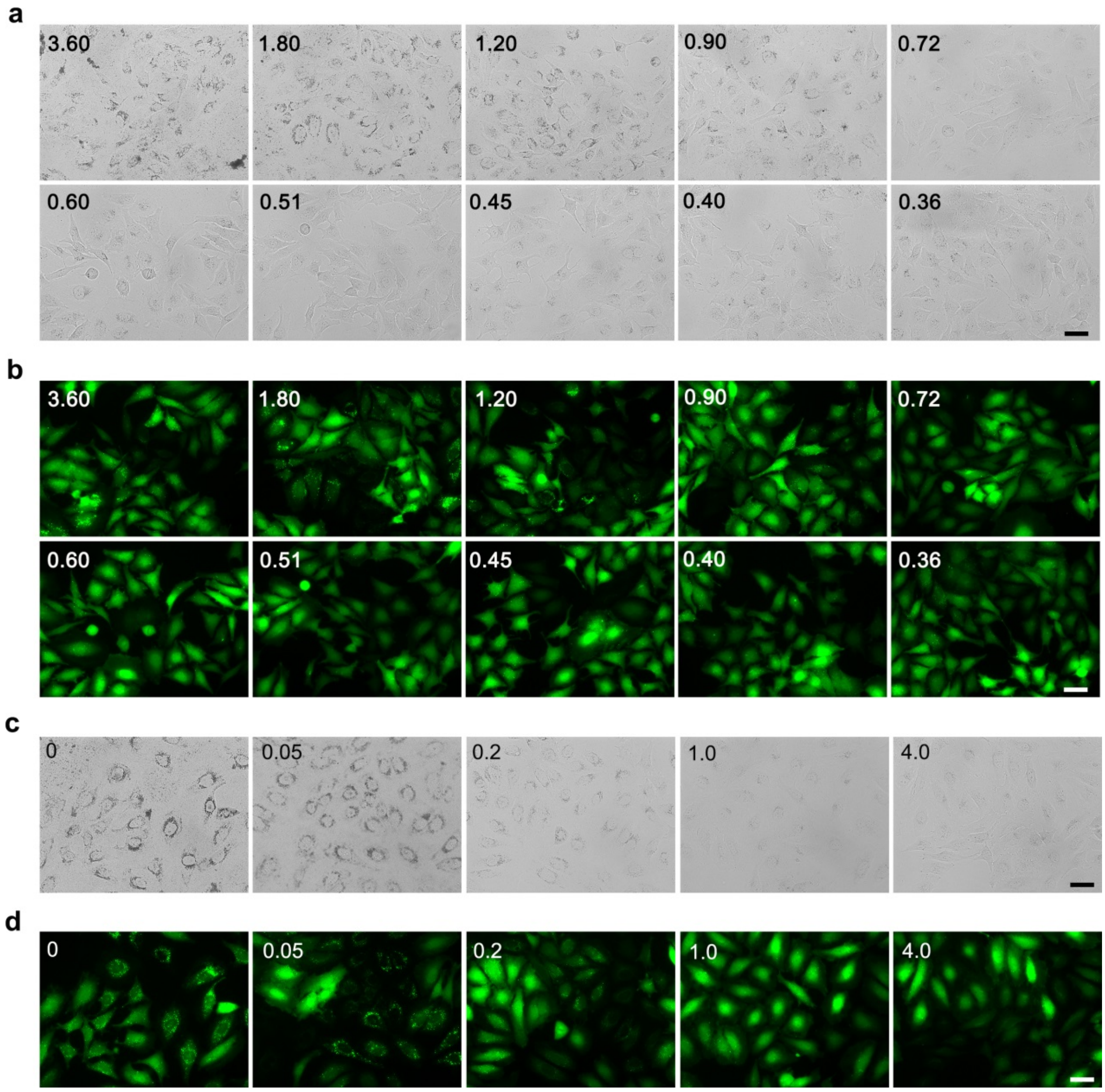

Supplementary Figure 10. Dilution and BSA adsorption improved dispersity and reduced autophagic effect. **a-b**, IO@citrate nanoparticles were added by no pipetting at different initial concentration (mg/mL). All of the final concentrations were 36 µg/mL. **c-d**, IO@citrate nanoparticles were dispersed in various concentration (mg/mL) of BSA solution before incubating with cells. The final concentration was 96 µg/mL of Fe and 120 µg/mL of BSA. All the scale bars were 50 µm.

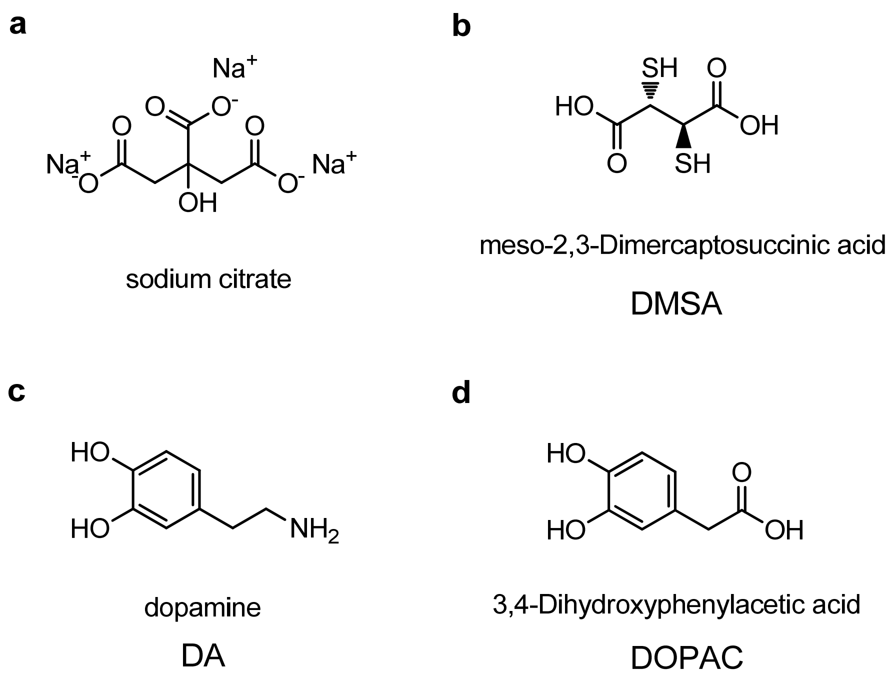

Supplementary Figure 11. Chemical structure of ligands on the surface of IO nanoparticles. **a**, Sodium citrate. **b**, DMSA. **c**, DA. **d**, DOPAC.

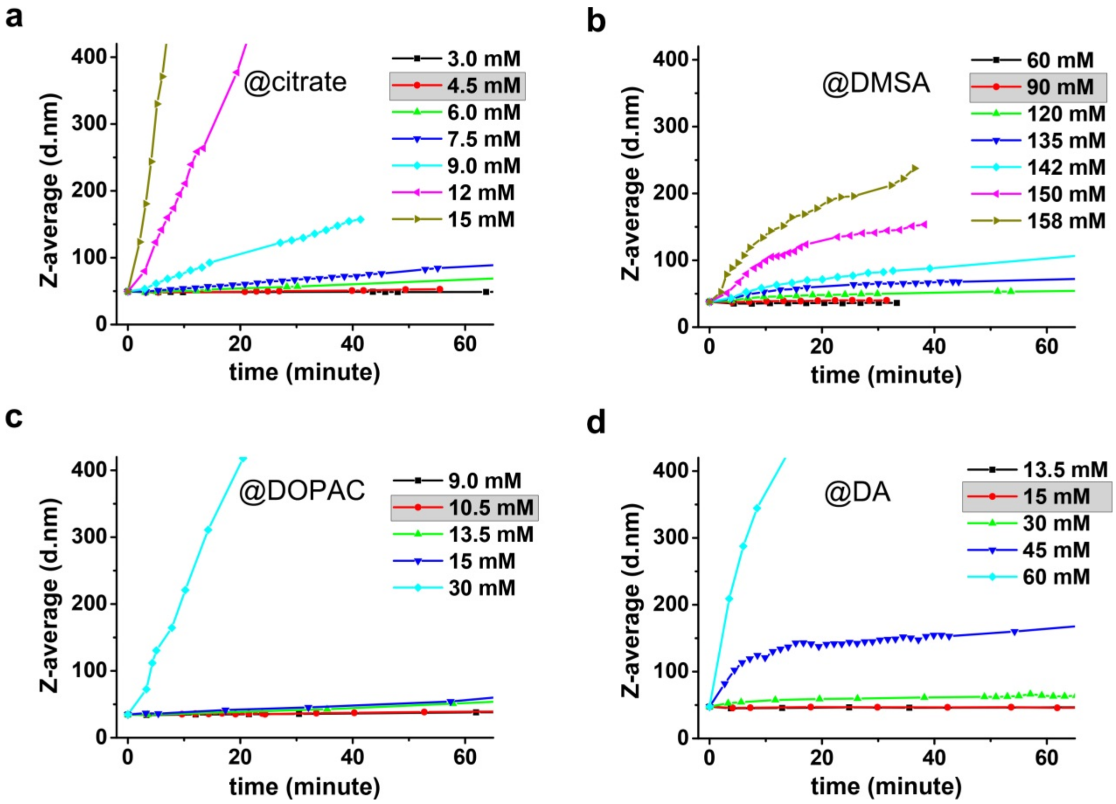

Supplementary Figure 12. Stability of IO nanoparticles with various coatings in saline. The max concentration of NaCl that IO nanoparticles kept stable was highlighted in each graph respectively. **a**, IO@citrate, 4.5 mM. **b**, IO@DMSA, 90 mM. **c**, IO@DOPAC, 10.5 mM. **d**, IO@DA, 15 mM.

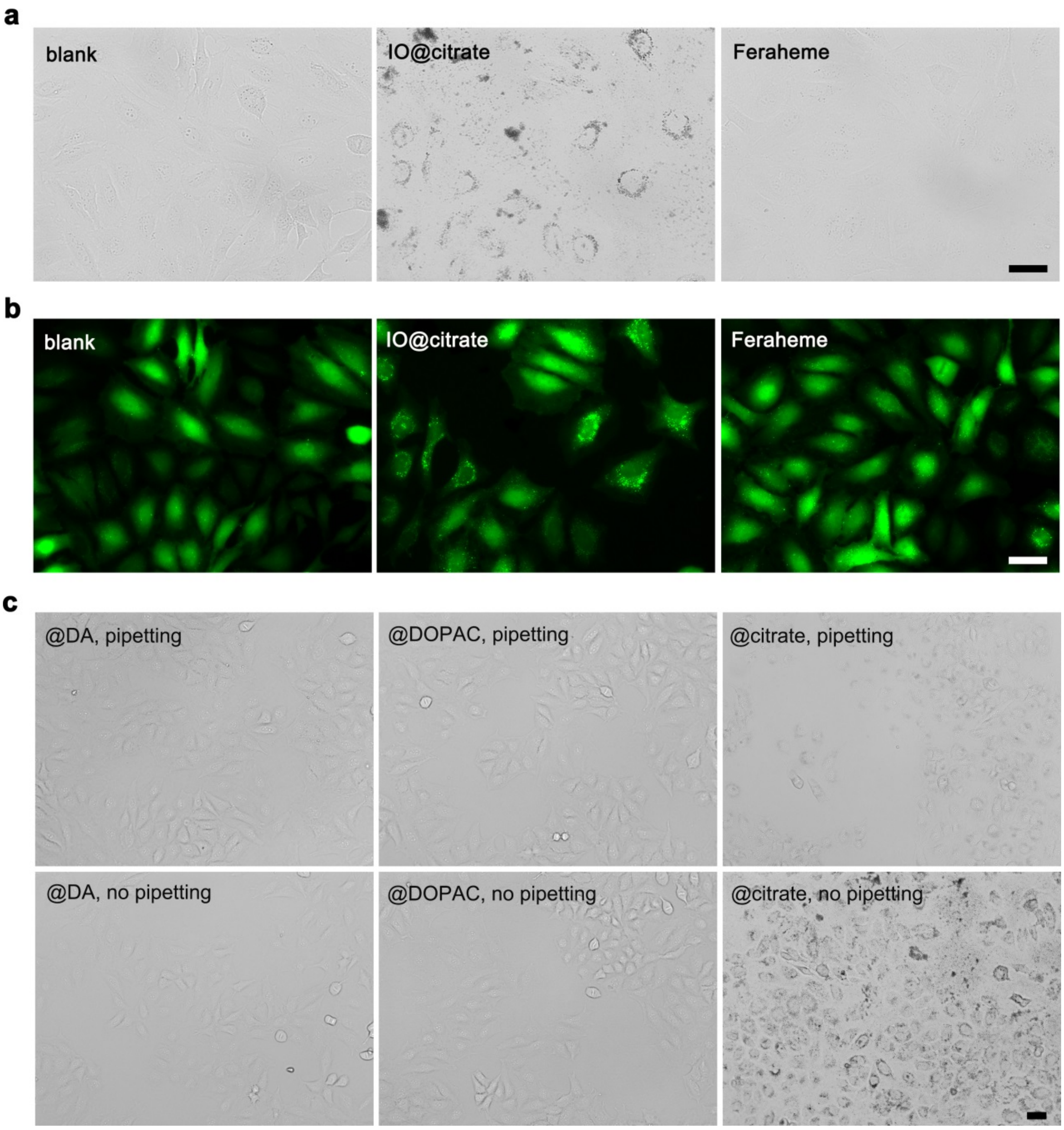

Supplementary Figure 13. Surface coating influenced the dispersity of IO nanoparticles. **a-b**, Commercial IO nanoparticles Feraheme (up to 300 μg/mL) dispersed well in complete medium, while IO@citrate nanoparticles (36 μg/mL) were aggregated and induced autophagic effect. **c**. IO@DA, IO@DOPAC nanoparticles were dispersed well in complete medium. They were added at the same initial (2.7 mg/mL) and final (107 μg/mL) concentration, by pipetting or not. Cells were treated for 24 h. All the scale bars were 50 μm.

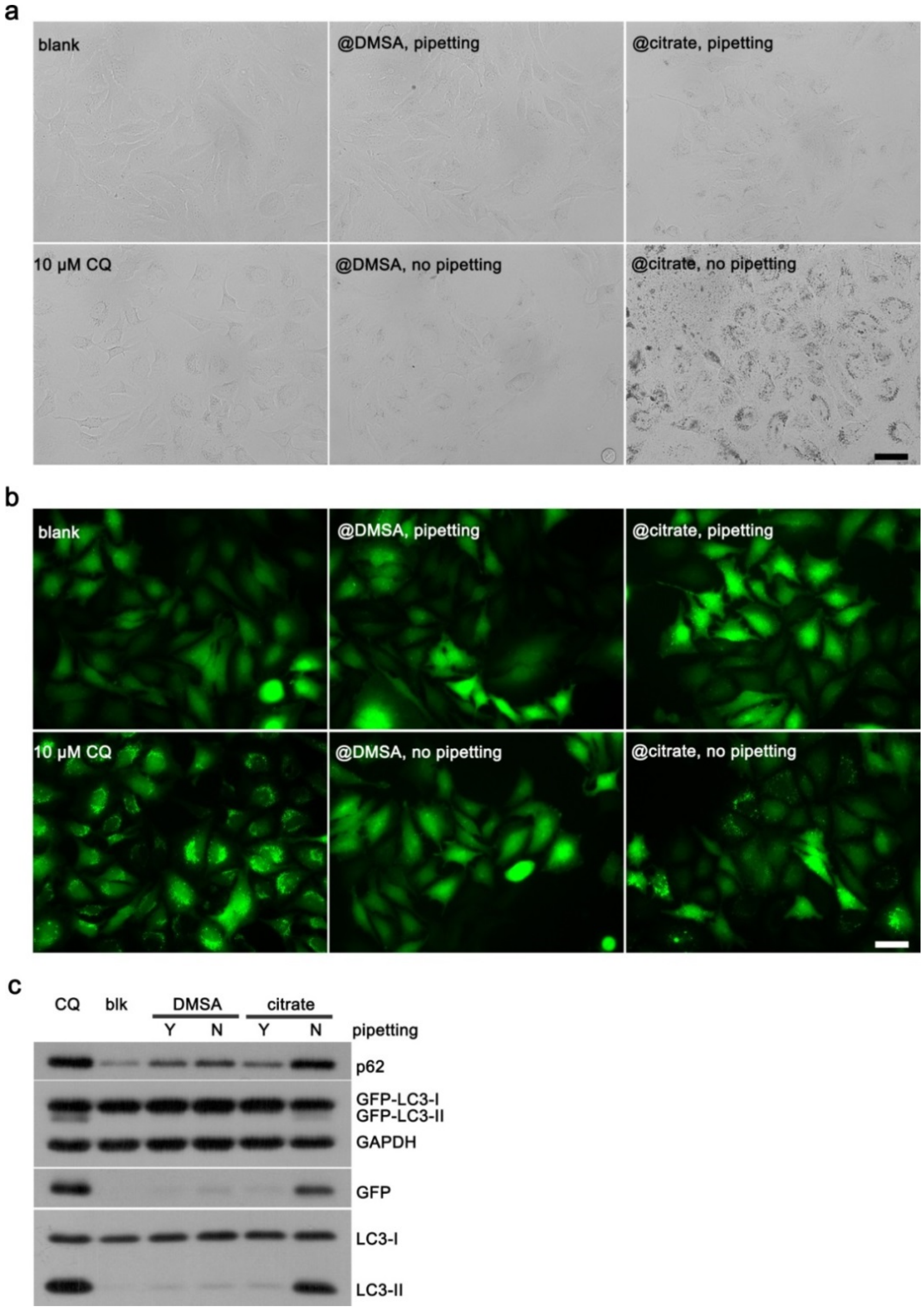

Supplementary Figure 14. IO@DMSA nanoparticles did not induced accumulation of autophagic vacuoles for better dispersity compared to IO@citrate nanoparticles. **a**, Bright field images. **b**, Fluorescence images of GFP-LC3. **c**, Western blot analysis of autophagy-related proteins. They were added at the same initial concentration (2.0 mg/mL) and final concentration (59 μg/mL). blk: blank. Cells were treated for 24 h. Scale bars were 50 μm.

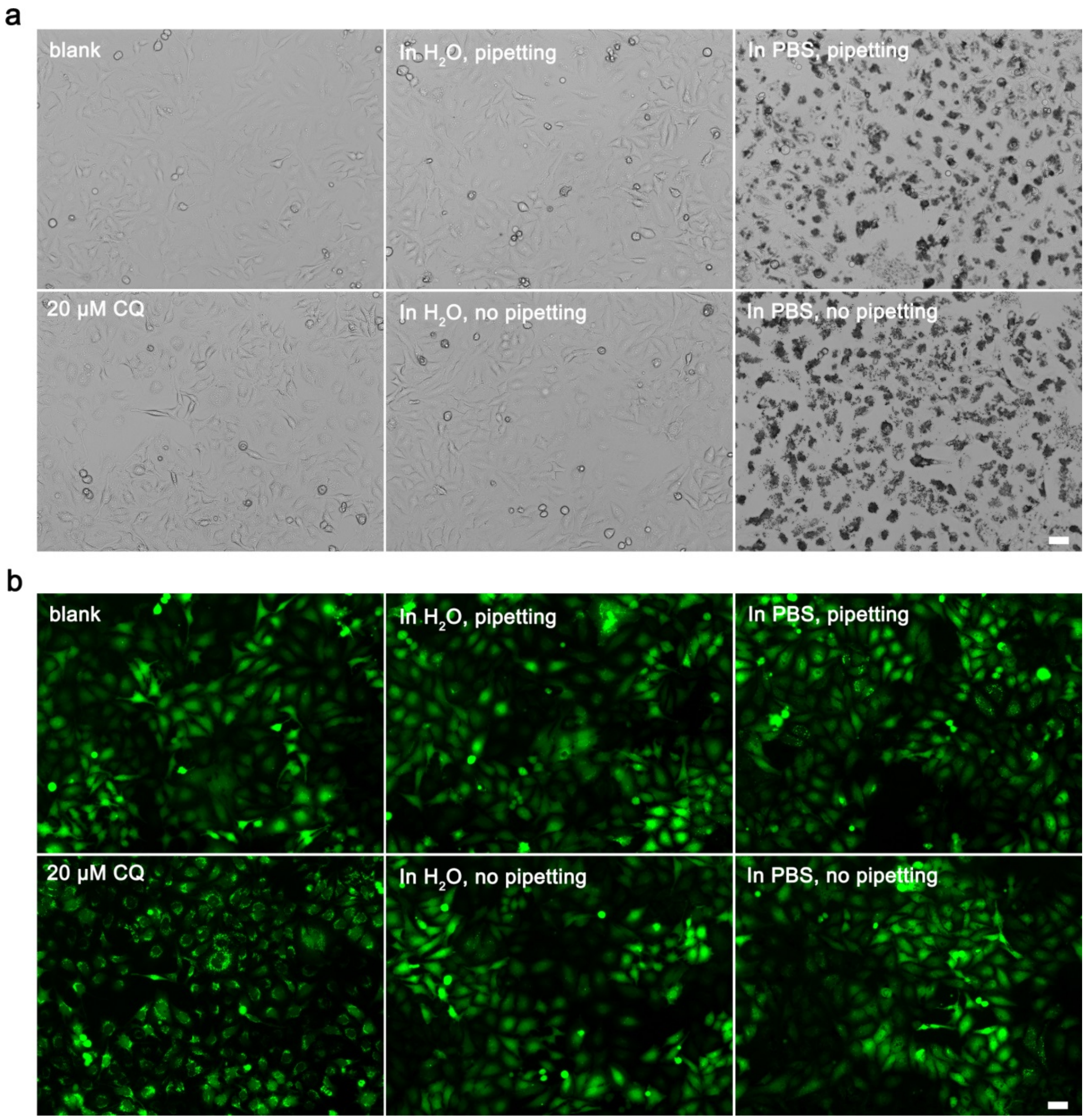

Supplementary Figure 15. IO@DA nanoparticles were aggregated and induced accumulation of autophagic vacuoles when dispersed in PBS. **a**, Bright field images. **b**, Fluorescence images of GFP-LC3. IO@DA nanoparticles were added at the same initial concentration (3.7 mg/mL) and final concentration (148 μg/mL). Cells were treated for 24 h. Scale bars were 50 μm.

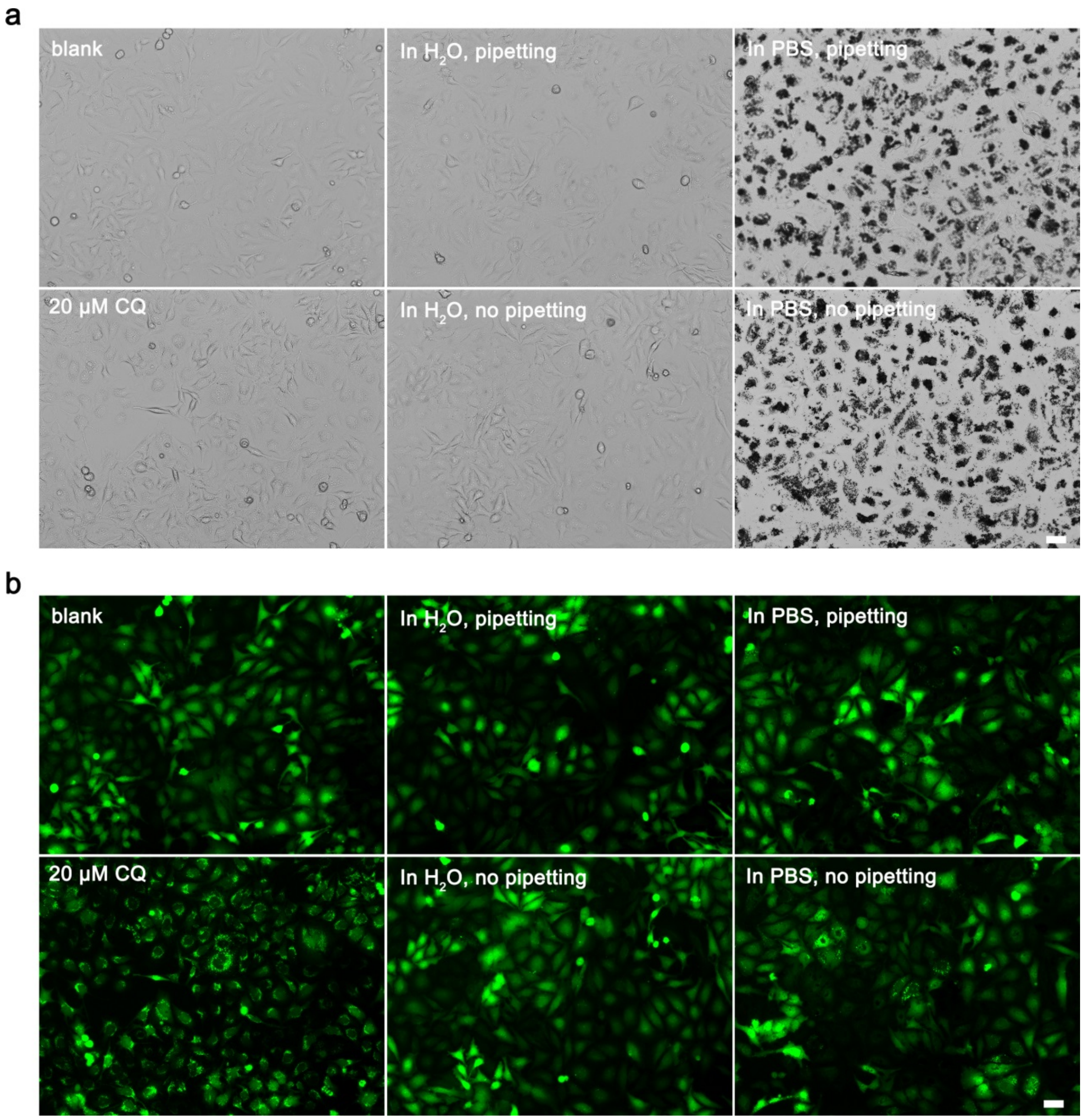

Supplementary Figure 16. IO@DOPAC nanoparticles were aggregated and induced accumulation of autophagic vacuoles when dispersed in PBS. **a**, Bright field images. **b**, Fluorescence images of GFP-LC3. IO@DOPAC nanoparticles were added at the same initial concentration (2.4 mg/mL) and final concentration (96 µg/mL). Cells were treated for 24 h. Scale bars were 50 µm.

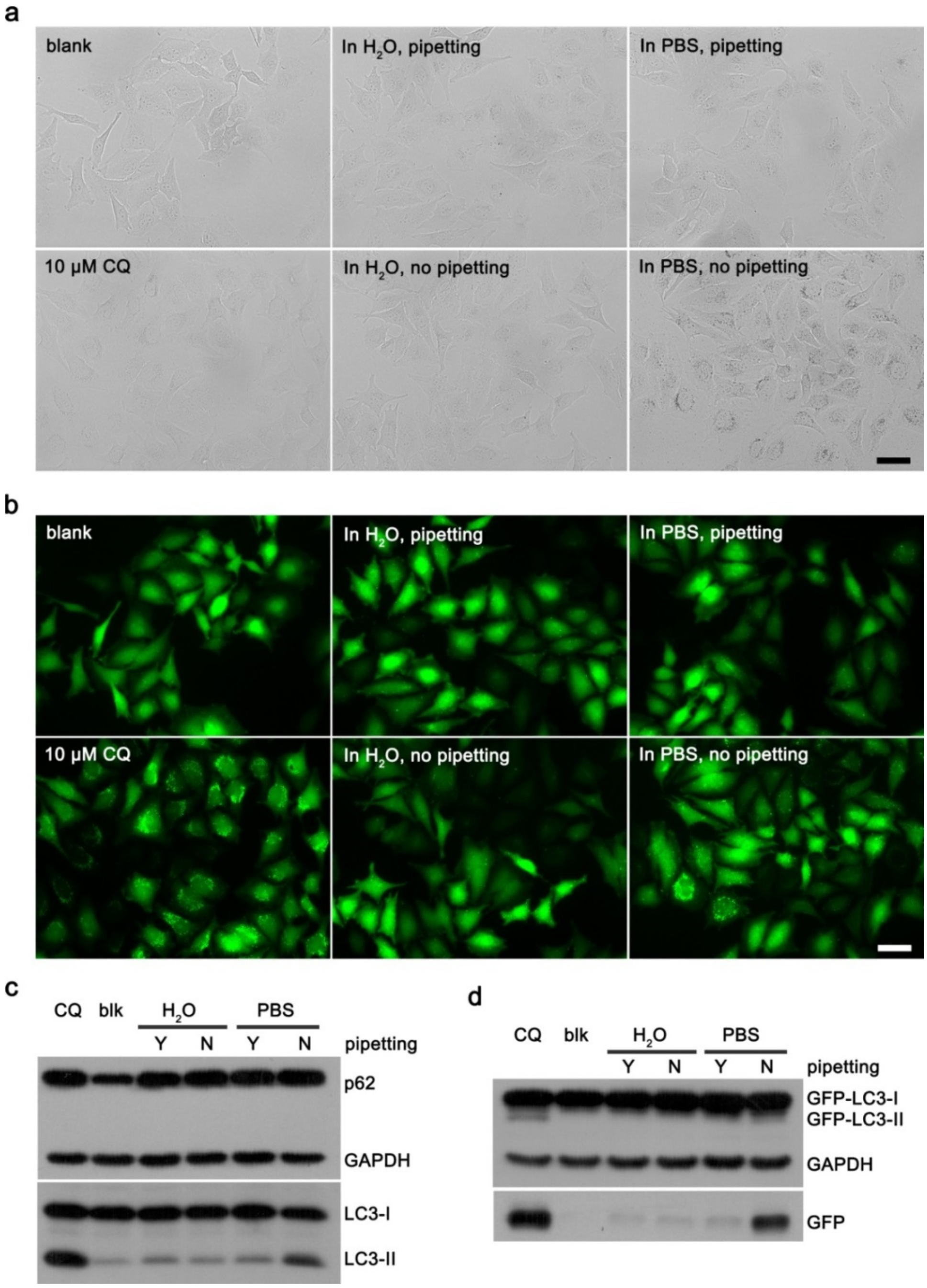

Supplementary Figure 17. IO@DMSA nanoparticles induced autophagic vacuole accumulation when aggregated. When dispersed in PBS, and mixed by no pipetting, IO@DMSA nanoparticles were aggregated. **a**, Bright field images. **b**, Fluorescence images of GFP-LC3. **c**, Western blot analysis of autophagy-related proteins. They were added at the same initial concentration (1.8 mg/mL) and final concentration (54 μg/mL). Scale bars were 50 μm.

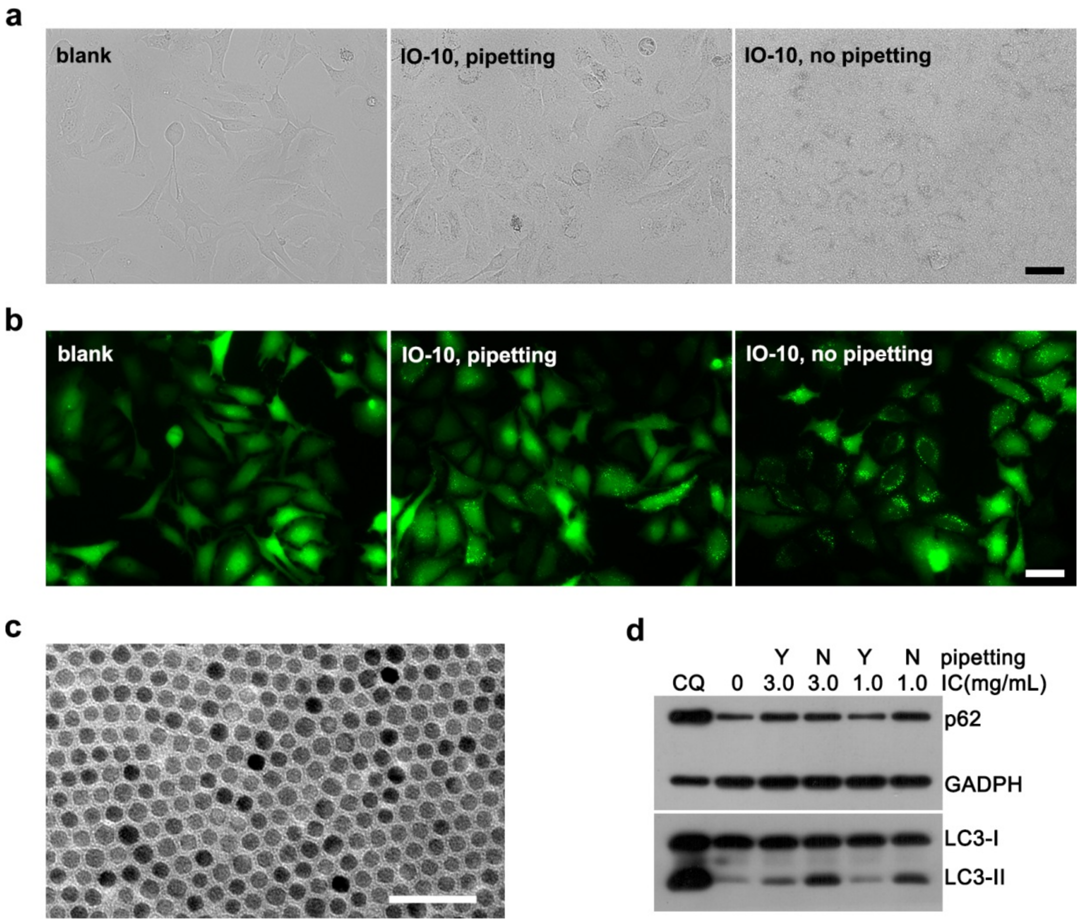

Supplementary Figure 18. 10 nm IO nanoparticles (IO-10) induced accumulation of autophagic vacuoles by dispersity-dependent. **a**, Bright field images. **b**, Fluorescence images of GFP-LC3. **c**, TEM images of IO-10. **d**, Western blot analysis of autophagy-related proteins. IC: Initial concentration. Cells were treated with IO-10 for 24 h. Scale bars were 50 μm (**a**, **b**) and 50 nm (**c**) respectively.

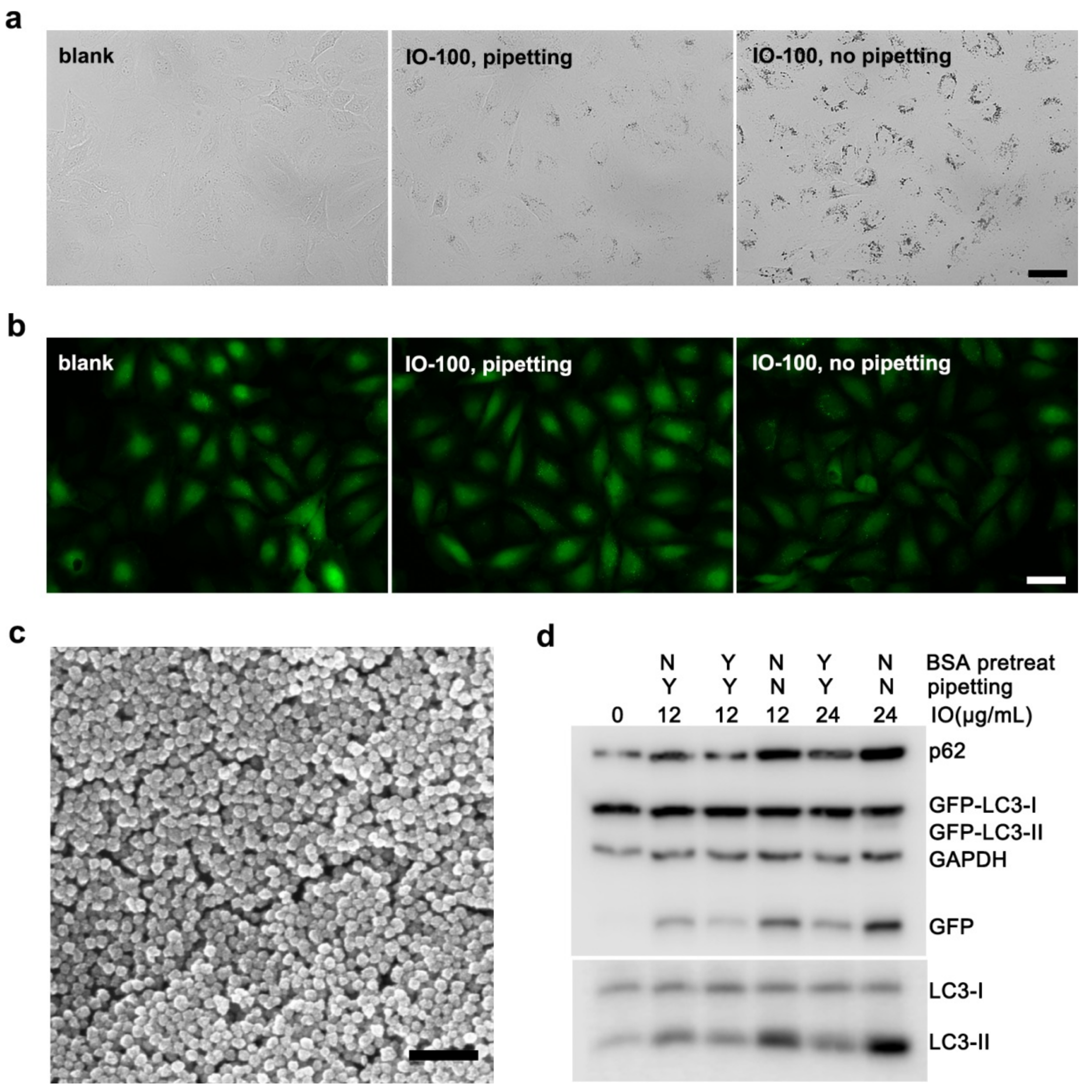

Supplementary Figure 19. 100 nm IO nanoparticles (IO-100) induced accumulation of autophagic vacuoles by dispersity-dependent. **a**, Bright field images. **b**, Fluorescence of GFP-LC3. **c**, SEM image of IO-100. **d**, Western blot analysis of autophagy-related proteins. Cells were treated with IO-100 for 24 h. Scale bars were 50 μm (**a**, **b**) and 500 nm (**c**) respectively.

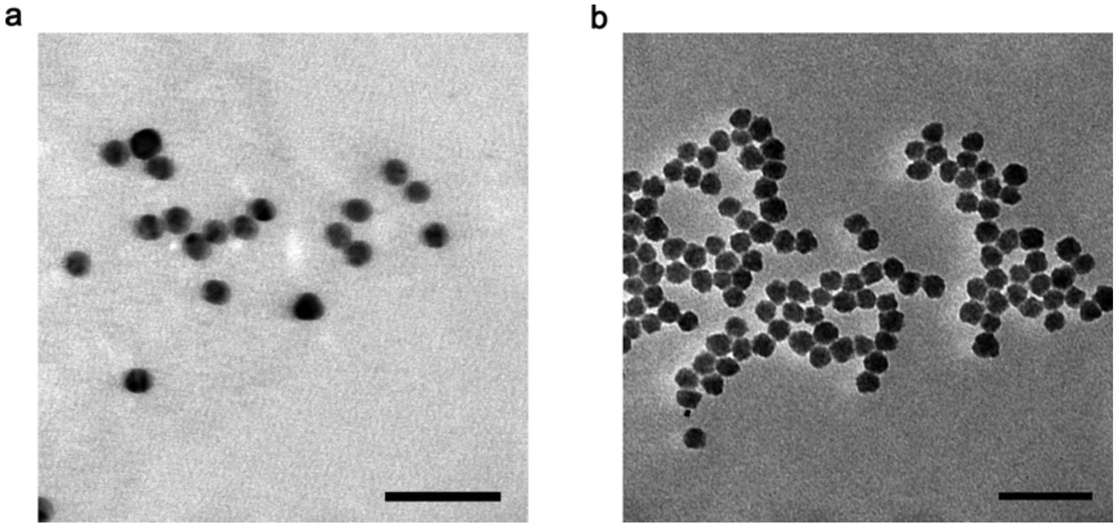

Supplementary Figure 20. TEM images of gold and silica nanoparticles. **a**, 12 nm gold nanoparticles. Scale bar was 50 nm. **b**, 28 nm silica nanoparticles. Scale bar was 100 nm.

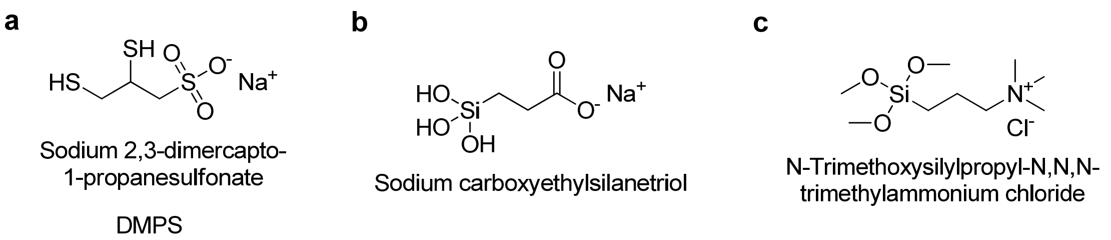

Supplementary Figure 21. Chemical structure of ligand on the surface of gold and silica nanoparticles. **a**, DMPS. **b**, Carboxylate. **c**, Quaternary ammonium cation.

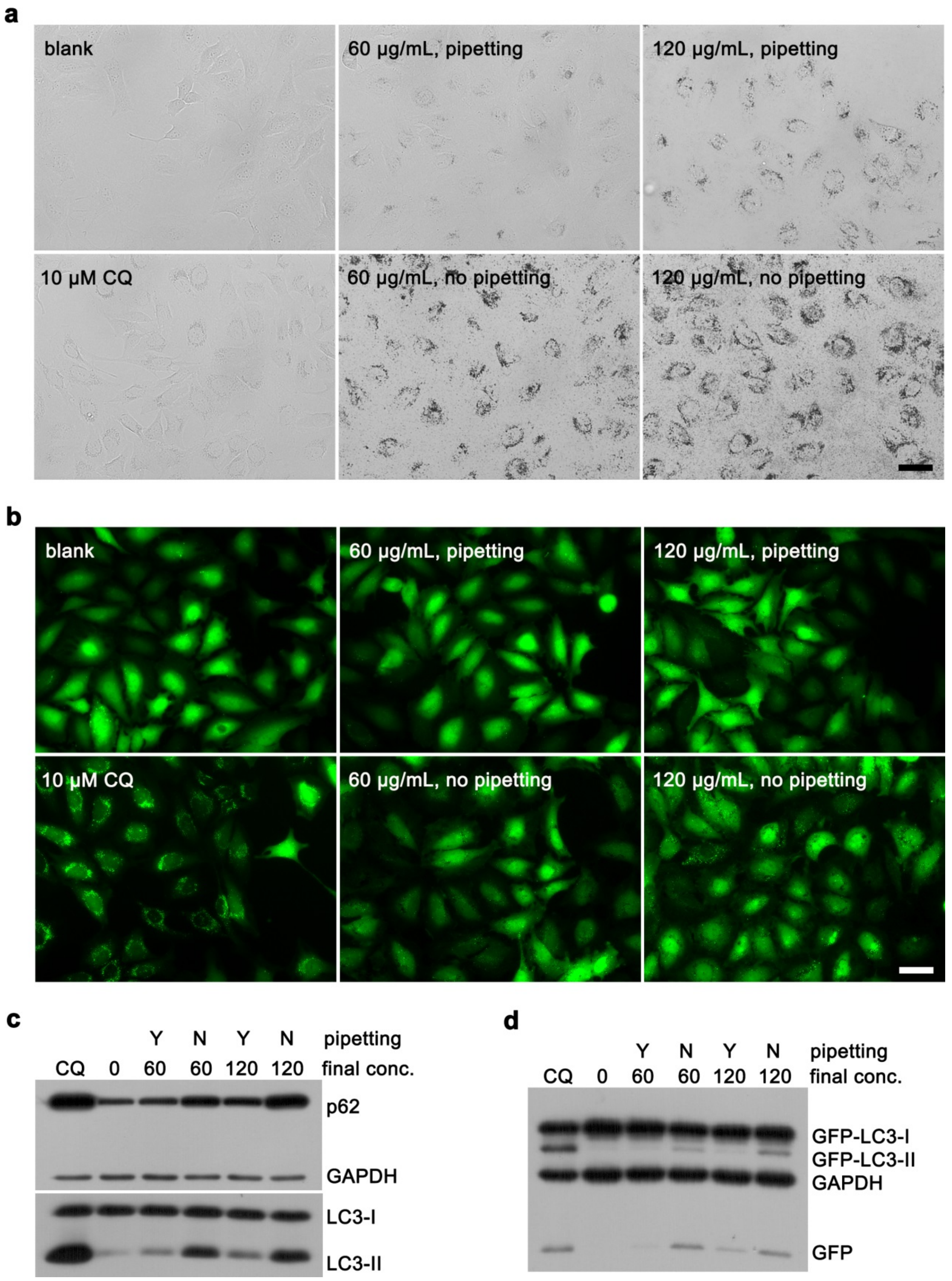

Supplementary Figure 22. Au@DMPS nanoparticles induced accumulation of autophagic vacuoles by dispersity-dependent. **a**, Bright field images. **b**, Fluorescence images of GFP-LC3. **c-d**, Western blot analysis of autophagy-related proteins. Scale bars were 50 μm.

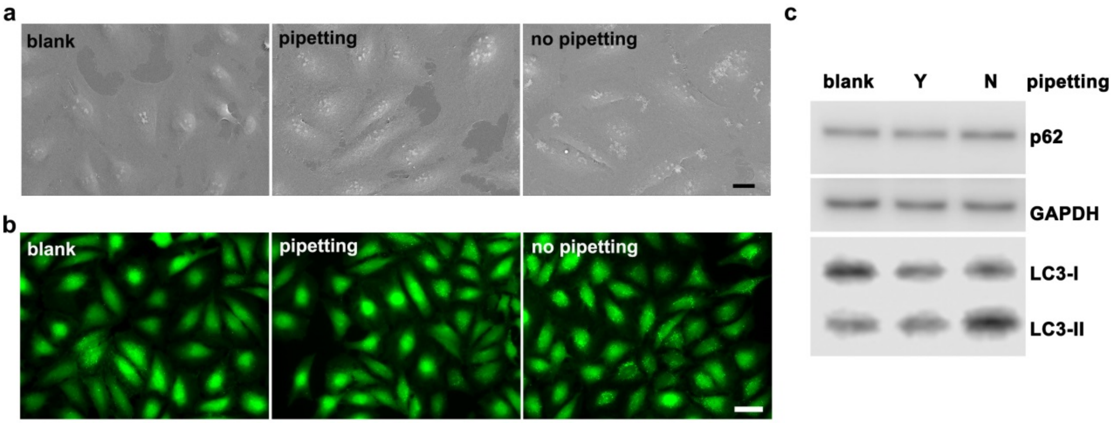

Supplementary Figure 23. SiO_2-QAC nanoparticles induced autophagic effect by dispersity-dependent. Cells were treated with SiO_2-QAC (16 μg/mL) for 24 h. **a,** SEM images. Scale bar was 20 μm. **b,** Fluorescence images of GFP-LC3. Scale bar was 50 μm. **c**, Western blot analysis.

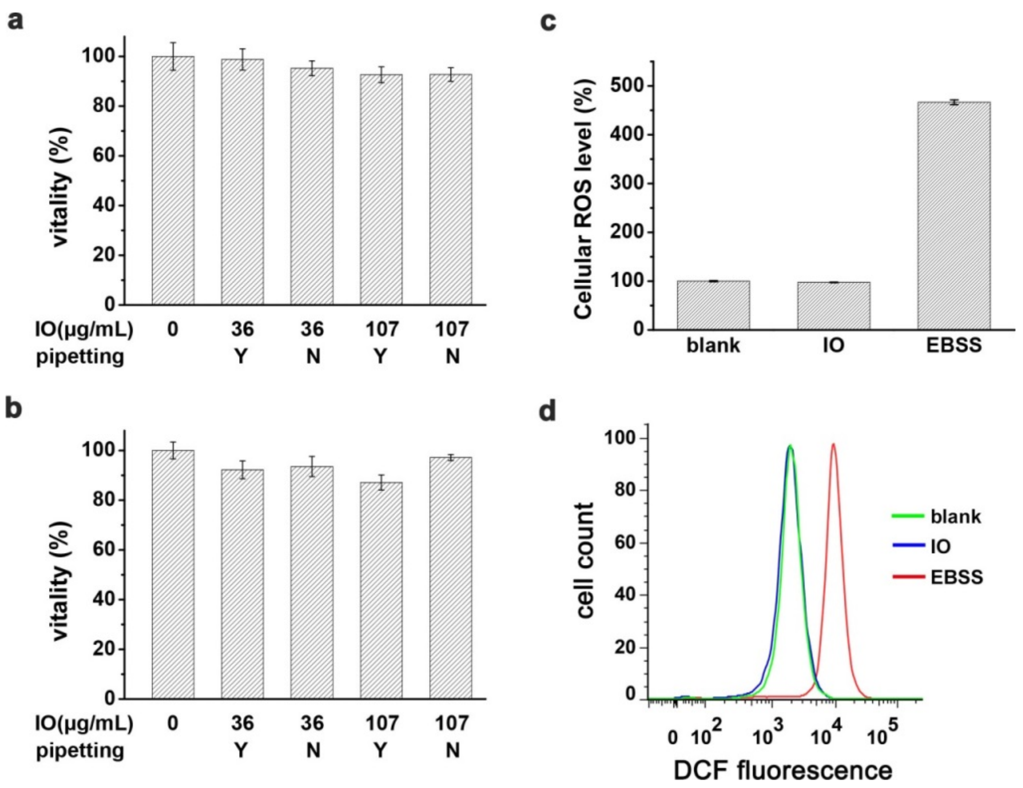

Supplementary Figure 24. Concurrent cellular responses to nanoparticles. Cytotoxicity of IO@citrate nanoparticles (n = 5. Data represent mean ± s.d.) at **a**, 24 h and **b**, 48 h. **c-d**, The ROS level of cells after treated with aggregated nanoparticles for 1 h.

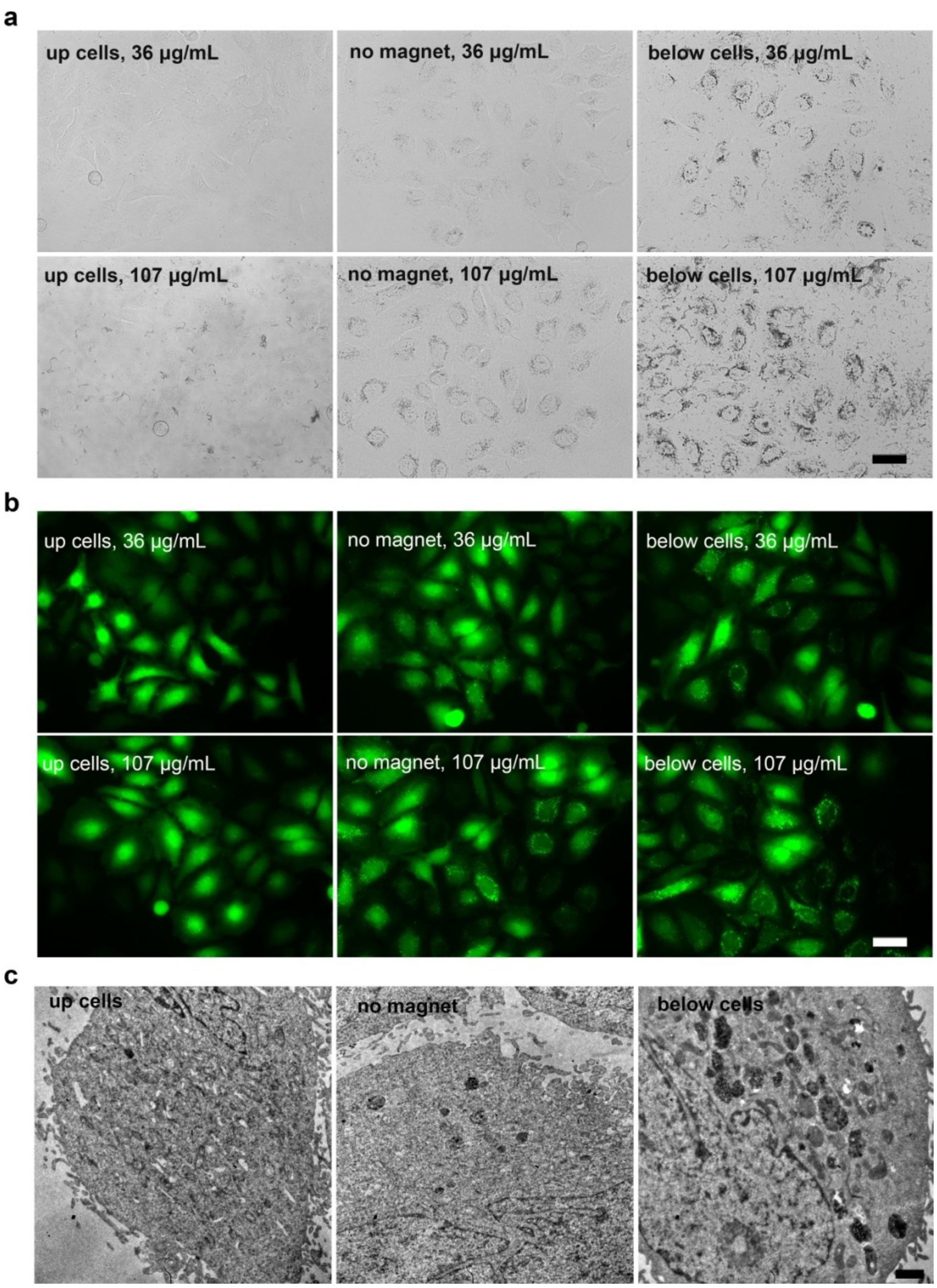

Supplementary Figure 25. Magnetic fields alter uptake level of IO@citrate nanoparticles and autophagic effect. Nanoparticles were mixed by pipetting. A NdFeB magnet was put up or below cells. **a**, Bright field images. **b**, Fluorescence images of GFP-LC3. **c**, TEM images of cells. Magnetic fields were 160 mT up cells and 330 mT below cells respectively. Scale bars were 50 μm (**a**, **b**) and 1 μm (**c**) respectively.

Supplementary Table 1. Size and zeta potential of IO nanoparticles with different coatings.

Coating	Size		Zeta potential	
	Z-Average(d.nm)	PdI	ζ(mV)	pH
citrate	48.9	0.164	-47.6	7.32
DMSA	37.8	0.154	-43.9	7.32
DA	47.6	0.137	-47.1	7.34
DOPAC	34.8	0.081	-47.0	7.32

Supplementary Methods

Synthesis of iron oleate complex

Iron-oleate was synthesized according to Park at el[1]. Briefly, 13.0 g $FeCl_3$ (80 mmol, CP, Sinopharm chemical), 73.0 g sodium oleate (240 mmol, chemical pure, CP, Sinopharm chemical) was dissolved in 160 mL ethanol and 80 mL ultrapure water (18.2 MΩ.cm, PURELAB flex 3, ELGA). The solution was heated to 70 °C and stirred for 4 h. After that, the upper layer containing iron-oleate was washed with water and then extracted by hexane (analytical reagent, AR, Sinopharm chemical). Finally, hexane was evaporated off, resulting in a semisolid iron oleate.

Synthesis of iron oxide nanoparticles

10 nm and 30 nm iron oxide nanoparticles (IO nanoparticles) were synthesized according to Park at el[1]. Briefly, 18 g iron oleate and 2.9 g oleic acid (90%, Alfa Aesar) was dissolved in 100 g 1-octadecene (90%, Alfa Aesar). The solution was degased at 100 °C for 60 min, and then heated to 320 °C with a rate of 3-4 °C/min. After refluxed for 60 min, the solution turned brown or black. Refluxed another 30 min, and cooled to room temperature. Nanoparticles were precipitated by adding ethanol and separated by centrifugation (5,000 x g, 3 min). The resulting IO nanoparticles were purified by re-dispersed in hexane and then precipitated in ethanol.

For synthesis of 10 nm IO nanoparticles (IO-10), 12.6 g iron oleate and 1.9 g oleic acid were dissolved in 60 g 1-octadecene. The solution was degased at 100 °C for 60 min, and then heated to 320 °C with a rate of 3-4 °C/min. Refluxed for 50 min and

cooled to room temperature. Separated and purified as above.

Surface modification of iron oxide nanoparticles with sodium citrate (IO@citrate)

58 mg citrate sodium dehydrate (AR, Sinopharm chemical) was dissolved in 8 g ultrapure water and 12 g acetone. 50 mg IO nanoparticles dissolved in 10 g hexane or cyclohexane (AR, Sinopharm chemical) was added. The solution was heated to 60 °C and refluxed for 8 h with a magnetic stir. After that, nanoparticles were coated with sodium citrate instead and transferred to water phase. Nanoparticles were separated by centrifugation (5,000 x g, 3 min), washed with acetone or methanol, dispersed in water and filtered by 0.22 μm filter. For further purification, IO@citrate nanoparticles were ultrafiltered (Amicon® Ultra-15, 100 kD, Millipore) for at least three times (10 mL liquid was concentrated to 0.5 mL each time) and finally filtered by 0.22 μm sterile filter (Millex-GP, Millipore) in class II biosafety cabinet (Steril GARD III, Berker) before used. Note: The amount of reagents is not strict for this process; The temperature can be higher; Acetone is necessary for ligand exchange.

Surface modification of iron oxide nanoparticles with dopamine (IO@DA)

35 mg dopamine hydrochloride (>98%, Sangon biotech) and 27 mg sodium hydroxide (AR, Sinopharm chemical) were dissolved in 20 g methanol (AR, Sinopharm chemical). 20 mg IO nanoparticles dissolved in 26 g cyclohexane was added and mixed. The solution was heated to 55 °C with a magnetic stir and kept for 6 h.

Separated and purified IO@DA nanoparticles similar to IO@citrate. Note: The amount of reagents is not strict for success; The temperature and time can be adjusted; nitrogen atmosphere is helpful for this process; Sodium hydroxide or other alkali (*e.g.*, sodium bicarbonate) is necessary for this process.

Surface modification of iron oxide nanoparticles with DOPAC (IO@DOPAC)

51 mg 3,4-Dihydroxyphenylacetic acid (DOPAC, 98%, Alfa Aesar) and 24 mg sodium hydroxide were dissolved in 23 g methanol, then added 30 mg IO nanoparticles dissolved in 26 g cyclohexane and mixed. The solution was heated to about 50°C with a magnetic stir and kept for 6 h. Separated and purified IO@DOPAC nanoparticles as above. Note: Refer above.

Surface modification of iron oxide nanoparticles with DMSA (IO@DMSA)

43 mg meso-2,3-dimercaptosuccinic acid (DMSA, 97%, Alfa Aesar) was dissolved in 21 g methanol, added 200 μL 1 M sodium hydroxide and 20 mg IO nanoparticles dissolved in 28 g cyclohexane. The solution was heated to 50-60 °C with magnetic stir and kept for 10 h. Separated and purified IO@DMSA nanoparticles as above. Note: Refer above.

Synthesis of 100 nm iron oxide nanoparticles coated with sodium citrate

The 100 nm iron oxide nanoparticles (IO-100) were synthesized according to Liu et al[2]. Briefly, 5.4 g (20 mmol) ferric chloride hexahydrate (>99%, Sigma-Aldrich), 1.47

g (5 mmol) citrate sodium dehydrate, was dissolved in 100 mL ethylene glycol (99.5%, Acros) by magnetic stir. Afterward, 10 g sodium acetate trihydrate was added and dissolved. The solution was sealed in a teflon-lined stainless-steel autoclave (200 mL capacity) and heated to 200 °C. After 13 h, stopped heating and allowed to cool to room temperature. The black products were washed with ultrapure water for three times and separated by a NdFeB magnet. IO-100 nanoparticles were filtered by 0.22 μm sterile filter before used.

Synthesis of gold nanoparticles

Gold nanoparticles were synthesized according to Turkevich method modified by Zakaria et al[3]. Briefly, 8 mL 50 mM chloroauric acid (ACS, 99.99%, Alfa Aesar) was added to 450 mL boiling ultrapure water, while kept heating and stirring. After one minute, 7.2 mL 250 mM sodium citrate was added. The solution soon turned blue, black, and then red. After 11 min, stopped heating and cooled to room temperature. The as-synthesized gold nanoparticles were about 12 nm and coated with sodium citrate.

Surface modification of gold nanoparticles with DMSA (Au@DMSA).

100 mL as-synthesized gold nanoparticles was diluted to 150 mL. 170 mg meso-2,3-dimercaptosuccinic acid (DMSA, 97%, Alfa Aesar) in 20 mL acetone or methanol was dropped into gold colloid solution at a rate of 250 μL/min while kept stirring. Reaction was kept for 6 h. Added NaCl to precipitated Au@DMSA and

collected by centrifugation (8,000 x g, 5 min). Then washed with 0.3% NaCl and dispersed in 4 mL ultrapure water. Au@DMSA nanoparticles were finally filtered by 0.22 μm sterile filter before used. Note: 10-20 mg DMSA is enough in this reaction; Adjusted pH to neutral or alkalescence is helpful for ligand exchange; It is important to add DMSA slowly at early stage.

Surface modification of gold nanoparticles with DMPS (Au@DMPS)

50 mL as-synthesized gold nanoparticles was diluted to 150 mL. Added 100 μL 0.5 M NaOH. Dropped 55 mg Sodium 2,3-dimercapto-1-propanesulfonate (DMPS, 95%, Aladdin) dissolved in 20 mL water at a rate of 250 μL/min. Afterward, kept stirring for 6 h. Collected and purified as Au@DMSA. The final volume was about 2 mL.

Synthesis of silica nanoparticles

The silica nanoparticles was synthesized according to Hartlen et al[4]. Briefly, 167 mg L-arginine (>98.5%, Sangon biotech) was dissolved in 135 mL ultrapure water in a 250 mL round flask. Then 9 mL cyclohexane was added. The solution was heated to 60 °C and kept stirring at 450 rpm. 45 mL tetraethoxysilane (TEOS, 99.9%, Alfa Aesar) was added. The reaction was kept for 30 h. The resulted silica nanoparticles were about 28 nm.

Surface modification of silica nanoparticles with carboxylate salt (SiO_2-COOH)

30 mL as-synthesized silica nanoparticles was diluted to 100 mL and heated to 60°C,

550 μL carboxyethylsilanetriol sodium (25% in water, J&K chemical) was added and the reaction was kept for 16 h and then cooled to room temperature. The solution was filtered by 0.22 μm filter before ultrafiltration (100 kD) for three times. The final volume was about 15 mL. The SiO_2@carboxylate nanoparticles was filtered by 0.22 μm sterile filter before used. Note: The reaction time could be shorted to about 4 h.

Surface modification of silica nanoparticles with quaternary ammonium cation (SiO_2-QAC)

15 mL as synthesized silica nanoparticles was diluted to 100 mL, added 1 mL acetic acid with ultrasonic, and then added 100 μL N-trimethoxysilylpropyl-N,N,N-trimethylammonium chloride (50% in methanol, J&K chemical). The solution was heated to 60 °C and kept for 13 h. Then cooled to room temperature. The solution was filtered by 0.22 μm filter before ultrafiltration (100 kD) for three times. The final volume was about 15 mL. The SiO_2-QAC nanoparticles was filtered by 0.22 μm sterile filter before used. Note: This protocol for modification of positive ligand need to be improved.

Determine the concentration of IO nanoparticles

We determined the concentration of IO nanoparticles by 1,10-phenanthroline spectrophotometry. 20 μL aqueous solution of IO nanoparticles were dissolved in 980 μL 1 M HCl and heated at 80 ℃ for 3 h (one hour was enough). Kept at room temperature for one day was also sufficient for digestion. Standard solution of 100

μg/mL Fe was prepared with $Fe(NH_4)_2(SO_4)_2.6H_2O$ (Mohr's salt) (AR, Sinopharm chemical). For each 5 mL tube, 800 μL 1 M sodium acetate, 300 μL 1 M HCl and 100 μL 10% hydroxylamine hydrochloride (AR, Sinopharm chemical) were added and mixed, followed by 100 μL sample or standard solution (0-100 μL, supplemented with 100-0 μL 1M HCl respectively). 300 μL 0.5% 1,10-phenanthroline (AR, Sinopharm chemical) was added for coloration. The result orange red solutions were stable at least for 6 hours at room temperature. The light absorption was measured at 510 nm. The R-Squared of calibration curve was usually larger than 0.995. All concentrations of IO nanoparticles were expressed as the concentration of iron element. This method is convenient and faster than ICP-AES. Additionally, Ferene S and Ferrozine are alternative colorimetric reagents of iron. Note: The concentration of 100 μL sample should be larger than 5 μg/mL; 1,10-phenanthroline should be dissolved in a little ethanol prior to in water.

Determine the concentration of gold nanoparticles

We determined the concentration of gold nanoparticles by ICP-AES. Gold nanoparticles were digested by aqua regia at 100 °C for 1 hour and continued for 12 h to decompose HNO_3. The residue was dissolved in 5% HCl for ICP-AES detection. All concentrations of gold nanoparticles were expressed as the concentration of gold element.

Determine the concentration of silica nanoparticles

We determined the concentration of silica nanoparticles by molybdenum blue spectrophotometry. 100 μL silica aqueous solution of silica nanoparticles were dissolved in 1 mL 2 M NaOH with a heating at 90 °C for 3 h. Standard solution of 1.0 mg/mL SiO_2 was prepared by sodium silicate with the same procedure. For each 5 mL tube, added standard solution (0-100 μL, supplemented with 100-0 μL 2 M NaOH respectively) or 100 μL sample. Acidified with 0.5 mL 1 M HCl, and then added 0.2 mL ethanol and 0.15 mL 5% ammonium molybdate (AR, Sinopharm chemical). Mixed and allowed reaction for 20 min at room temperature until the mixture turned yellow. Reduced it with 0.1 mL 5% vitamin C (AR, Sinopharm chemical). The solution turned blue within 5 min. Finally added 1.5 mL 3 M HCl and mixed. The resulted solution was stable at least 2 h at room temperature. The light absorption was measured at 650 nm. All concentrations of silica nanoparticles were expressed as the concentration of SiO_2. Note: The concentration of 100 μL sample should be larger than 50 μg/mL.

Cell culture

HeLa, SKOV3 and 293T cells were purchased from cell bank of Chinese Academy of Sciences. Cells were cultured in various medium (Hyclone) supplemented 10% fetal bovine serum (FBS, from Australia, Gibco) as follows: HeLa, Minimum Essential Medium (MEM); SKOV3, McCoy's 5A; 293T, Dulbecco's Modified Eagle Medium (DMEM). All the cells were maintained at 37 °C, 5% CO_2 (Thermo forma series II).

Construction of SKOV3 cell lines stably express GFP-LC3 (SKOV3-GFP-LC3)

SKOV3 cells were seeded in 96-well microplate (Nunc) at a density of 1.3×10^4/well. When cells were 70% -90% confluent next day, added the mixture of pSELECT-GFP-LC3 plasmid (Invivogen) and lipofectamine 2000 (Invitrogen, Life Tech) and incubated for about 4 h. Changed the medium and incubated for another 24 h. The desired cells were screened by 600 μg/mL zeocin (Invivogen). One or two weeks later, picked out cell clones that stably expressed GFP-LC3 using fluorescence microscope (Axio Observer A1, Zeiss). Kept cell clones stressed using zeocin until one clone grew up to about ten millions that enough for cryopreservation. Afterward we did not add zeocin to kept stress. Note: The ratio of lipofectamine and plasmid should be varied for optimized result; For detail transfection protocol, refer to the manufacture's guide by Life Tech; One may need try many times to get stable cell lines using plasmid transfection.

Construction of HeLa cell lines stably express GFP-LC3 (HeLa-GFP-LC3)

Lentivirus is highly efficient to integrate exogenous gene into host cells[5]. The lentivirus vector containing GFP-LC3 gene was built with the help of Biowit Technologies. We produced lentivirus in 293T cells using lentiviral packaging kit (Biowit Technologies). Lentivirus could be concentrated by ultracentrifugation or polyethylene glycol 6000[6]. Various amount lentivirus was added to HeLa cells in logarithmic phase in six groups. 8 μg/mL protamine was added to help infection. 12 h later, refreshed the medium and incubated for two days. (Note: All operation about

lentivirus should be performed in class II biosafe cabinet. All materials that touch lentivirus should be sterilized.) Picked out groups that more cells expressed GFP-LC3 using fluorescence microscope. Cells were screened by 2.5 μg/mL puromycin (>98%, Sangon Biotech). One week later, cell clones that mostly expressed GFP-LC3 were picked out using fluorescence microscope. These cells were diluted by series in 96-well microplate. Marked wells that had only one cell. About 10% single-cell clone would grow up to ten millions one month later. Picked out cells that grown well and had equal expression of GFP-LC3. After finishing construction, we did not add any puromycin. HeLa-GFP-LC3 cells were maintained with MEM supplemented 10% FBS as well.

Western blot

One could get detail and useful guides of western blot for free charge from Abcam, Bio-Rad, Millipore, Cell Signaling Technology and Pierce. We performed western blot according to these guides with some modifications.

Preparation of protein sample. After treated, cells in 6-well plate or 3.5 cm culture dish (Nunc) were collected by trypsin (Hyclone) and centrifuged to a pellet. Radio immune precipitation assay (RIPA) lysis buffer (Pierce) containing 1% protein inhibitor cocktail (Sigma-Aldrich) were added in ice bath and mixed with vortex and ultrasound. Cell pellet was continued lysing for about 30 min in ice bath. Centrifuged (15,000 x g, 4 °C, 5 min), collected the supernate and mixed with isometric Laemmli 2x loading buffer. The mixture was heated 3-5 min in boiling water to degenerated

protein and then cooled to room temperature. The protein sample that ready for western blot was stored at -20 °C or -80 °C for long term.

SDS-PAGE. Electrophoresis was performed using Mini-PROTEAN Tetra Cell (Bio-Rad). 10%-12% separating gel was suitable for separation of LC3-I and LC3-II; 8% was suitable for GFP-LC3-I and GFP-LC3-II; 6% gel was needed for mTOR detection. Loaded 10 μg protein each lane in 10-well gel or 6 μg in 15-well gel. Prestained color protein marker (10-170 kD, Pierce, #26616) was loaded to visualize protein ladder and protein transferring. The voltage was 10 V/cm when samples were in stacking gel and 15 V/cm in separating gel.

Transferring and blocking. Protein was transferred from gel to 0.45 μm polyvinylidene difluoride (PVDF) membrane (Immobilon-P, Millipore) by semi-dry-transfer (Trans-Blot Turbo Transfer System, Bio-Rad). PVDF membrane was blocked with 5% nonfat milk in tris-buffered saline (TBS) buffer for 1 hour.

Antibody staining. All the primary antibodies were produced in rabbit. Phosphospecific antibodies were diluted with 5% BSA in TBS and others were diluted in blocking buffer. Antibodies were listed as follows: Anti-LC3B (1/3,000, Abcam, ab51520), Anti-GAPDH (1/10,000, Abcam, ab128915), Anti-SQSTM1/p62 (1/10,000 – 1/20,000, Abcam, ab109012), Anti-GFP (1/5,000, Abcam, ab137827), Anti-LAMP1 (1/1,000, Cell Signaling Technology, #9091, D2D11 XP). Anti-mTOR (1/1,000, Abcam, ab134903), Anti-p-mTOR (1/1,000, S2481, Abcam, ab137133), Anti-β-tubulin (1/5,000, Abcam, ab68193). PVDF membrane was incubated with primary antibody for 2 h at room temperature or overnight at 4 °C for phosphospecific

antibodies, and washed with TBS buffer for 5 min x 5 times. Then incubated with Goat polyclonal Secondary Antibody to Rabbit IgG – H&L (horse radish peroxidase, HRP) (1/5,000 – 1/10,000, Abcam, ab97051) for 1 hour at room temperature and washed with TBS buffer for 5 min x 5 times.

Signal development. Added enhanced chemiluminescence (ECL) HRP substrate (WesternBright, Advansta) to membrane in a transparent plastic wrap and incubated for 5 min. Acquired images using X-Ray film (Kodak) in dark-room or scanned the membrane using chemiluminescence imaging systems (C-DiGit, Licor).

Buffer. 20 x TBS: 48.4 g Tris base (>99.9%, Sangon Biotech), 160 g NaCl (AR, Sinopharm chemical), adjusted pH to 7.4 (25 °C) with HCl (AR, Sinopharm chemical), added water to 1 L. Diluted 50 mL concentrated TBS to 1 L before used. 1 x transferring buffer: 2.9 g Tris base, 5.8 g glycine (>99%, Sangon Biotech), 0-200 mL methanol, supplemented water to 1 L.

Immunofluorescence staining

We performed immunofluorescence staining mainly according to the protocol from Cell Signaling Technology. Cells were seeded in glass-bottom (0.14 –0.18 mm) culture dish (Nest) so that they could be observed in oil immersion objective.

Fixation and blocking. After treated, cells were fixed with 4% paraformaldehyde (>95%, Sangon Biotech) in PBS for 15 min at room temperature. Washed with PBS, 5 min x 3.

Permeabilization. Permeabilized cells with cooled methanol at -20 °C for 10 min.

washed with PBS. It is optional.

Blocking. Blocked cells in PBS with 10% goat normal serum (New Zealand origin, Gibco) and 0.3% Triton X-100 (Sigma-Aldrich) for one hour.

Primary antibody staining. Anti-LC3B (1/200, Cell Signaling Technology, D11 XP), Anti-SQSTM1/p62 (1/200, Abcam, ab109012), Anti-LAMP1 (1/1,100, Cell Signaling Technology, #9091, D2D11 XP) antibody was diluted in antibody dilution buffer (1% BSA and 0.3% Triton X-100 in PBS). Incubated overnight at 4 °C. Washed with PBS for 5 min x 5.

Secondary antibody staining. Goat Anti-Rabbit IgG (H+L), DyLight 633 Conjugated (1/200 - 1/500, Pierce, Thermo scientific) antibody was diluted in antibody dilution buffer and incubated with cells at room temperature for 1 hour. Washed with PBS for 5 min x 5.

Images were acquired using laser scanning confocal microscope (Leica TCS SP5).

Scanning electron microscopy (SEM) imaging of cells

This is an intuitive and reliable method to distinguish nanoparticles inside cells from that is just attached on the surface. Cells were fixed in situ with 2% glutaraldehyde (electron microscopy grade, EM grade, SPI Supplies) in PBS for 15 – 30 min at room temperature. Washed with PBS for 5 min x 4. Further fixed with 1% osmium tetroxide (EM grade, SPI Supplies) in PBS for 1 hour at 4 °C. (Note: Osmium tetroxide is highly toxicity. Handle it in fume hood and collect the waste specially.) Washed with PBS for 5 min x 4. Dehydrate cells by ethanol with increasing concentration (30%,

50%, 70%, 90%, 100%, respectively) for 5-10 min each time. Repeated twice in 100% ethanol and then replaced it with tert-butanol for 15 min x 3. After that, dried it under vacuum or dryer. Coated cell with about 13 nm Pt using auto fine coater (JFC-1600, JEOL). Images were acquired using Zeiss Sigma SEM.

Transmission electron microscopy (TEM) imaging of cells

Cells were collected by trypsin and centrifuged (400 x g, 4 min) to a pallet. Fixed with 2% glutaraldehyde (EM grade, SPI Supplies) at 4 °C overnight. Washed with PBS for 5 min x 5. Further fixed with 1% osmium tetroxide (EM grade, SPI Supplies) for 1 hour at 4 °C. (Note: Osmium tetroxide is highly toxicity. Handle it in fume hood and collect the waste specially.) Washed with PBS for 5 min x 4. Dehydrate cells in ethanol with increasing concentration (30%, 50%, 70%, 90%, 100%, respectively) for 5-10 min each time. Repeated once in 100% ethanol and then replaced it with acetone for 5 min x 2. Cell pallet was embedded in SPI-Pon 812 resin (SPI Supplies). Polymerized at 35 °C for 12 h, and 60 °C for 24 - 48 h. Sectioned samples (60-80 nm) at ultramicrotome (Leica Ultracut). Sections were stained with 3 % uranyl acetate (EM grade, SPI Supplies) and lead citrate (EM grade, SPI Supplies). TEM images were acquired in JEM-1400 (JEOL), 100 kV.

Dynamic light scattering (DLS) measurement

Hydrodynamic diameter and zeta potential of colloid nanoparticles was measured using dynamic light scattering instrument (Nano-ZS90, Malvern). Light source is

He-Ne laser, 633 nm, 10 m W. Measurement angel is 90°.

Measurement temperature was set as equal to or slightly higher than the temperature of solvent at room temperature. To measure the stability of colloid nanoparticles (hydrodynamic diameter over time), measurement number was set to 30 or higher. Results of size measurement were presented as Z-Average and polydispersity index (PdI). Z-Average is average diameter of intensity-weight distribution. Note: It is very important to use highly pure water and cleaning pipette.

For zeta potential measurement, IO nanoparticles were dispersed in 2 mM phosphate buffer with various pH (varied ratio of 2 mM monosodium phosphate and 2 mM disodium phosphate). The pH of silica nanoparticles was adjusted by HCl and NaOH.

Flow cytometry measurement

Cells were collected by trypsin after treatment and staining. After that, cells were placed in ice bath until measured. Data was acquired in BD FACSAria II cytometry. 10,000 events were record for analysis. The first sample would be re-measured at the end of measurement to confirm the stability of signal.

Cyto-ID Autophagy Detection Kit (Enzo Life Science) was used to measure autophagic vacuoles. We performed that according to the instruction manual from manufacturer. To avoid interference, we used HeLa cells that did not expressed GFP-LC3.

Magic Red Cathepsin B Assay Kit (ImunoChemistry) was engaged to evaluate the activity of cathepsin B, a lysosomal cysteine protease. We performed it on HeLa cells

that did not expressed GFP-LC3, according to the manufacture's guide.

2′,7′-Dichlorofluorescin diacetate (DCFH-DA, Sigma, D6883) was engaged to measure cellular ROS level. After treatment, cells were staining with 50 μM DCFH-DA for 30 min at 37 °C. Then cells were collected and re-suspended in HEPES (4-(2-Hydroxyethyl)piperazine-1-ethanesulfonic acid, Sangon Biotech) buffer or PBS buffer.

Methyl thiazolyl tetrazolium (MTT) assay

Cells were seed in 96-well plate at a density of 3 x 10^3/well (for two days) or 5 x 10^3 /well (for one day). 12 h or 24 h later, replaced by the fresh medium containing nanoparticles. We set six parallel wells each group. After 24 h or 48 h, five of the parallel wells were refreshed by medium containing 0.5 mg/mL MTT. To eliminate the absorption of cells and nanoparticles, we did not add MTT into the sixth well. 4 h later, discarded the medium and replaced with 100 μL dimethylsulfoxide (DMSO). Shook for 5 min and measured absorption at 490 nm or 570 nm on microplate reader (Multiskan FC, Thermo Scientific). Note: In most cases, there is no difference between measuring at 490 nm and 570 nm.

Supplementary References

1. Park, J. *et al.* Ultra-large-scale syntheses of monodisperse nanocrystals. *Nature Mater.* **3,** 891-895 (2004).
2. Liu, J. *et al.* Highly water-dispersible biocompatible magnetite particles with low cytotoxicity stabilized by citrate groups. *Angew. Chem.* **121,** 5989-5993 (2009).
3. Zakaria, H. M. *et al.* Small molecule- and amino acid-induced aggregation of gold nanoparticles. *Langmuir* **29,** 7661-7673 (2013).
4. Hartlen, K. D., Athanasopoulos, A. P. T. & Kitaev, V. Facile preparation of highly monodisperse small silica spheres (15 to >200 nm) suitable for colloidal templating and formation of ordered arrays. *Langmuir* **24,** 1714-1720 (2008).
5. Sakuma, T., Barry, M. A. & Ikeda, Y. Lentiviral vectors: Basic to translational. *Biochem. J* **443,** 603-618 (2012).
6. Kutner, R. H., Zhang, X.-Y. & Reiser, J. Production, concentration and titration of pseudotyped HIV-1-based lentiviral vectors. *Nature Protoc.* **4,** 495-505 (2009).
